# Single-cell copy number calling and event history reconstruction

**DOI:** 10.1093/bioinformatics/btaf072

**Published:** 2025-02-13

**Authors:** Jack Kuipers, Mustafa Anıl Tuncel, Pedro F Ferreira, Katharina Jahn, Niko Beerenwinkel

**Affiliations:** Department of Biosystems Science and Engineering, ETH Zurich, Basel 4056, Switzerland; SIB Swiss Institute of Bioinformatics, Basel 4056, Switzerland; Department of Biosystems Science and Engineering, ETH Zurich, Basel 4056, Switzerland; SIB Swiss Institute of Bioinformatics, Basel 4056, Switzerland; Department of Biosystems Science and Engineering, ETH Zurich, Basel 4056, Switzerland; SIB Swiss Institute of Bioinformatics, Basel 4056, Switzerland; Department of Biosystems Science and Engineering, ETH Zurich, Basel 4056, Switzerland; SIB Swiss Institute of Bioinformatics, Basel 4056, Switzerland; Department of Biosystems Science and Engineering, ETH Zurich, Basel 4056, Switzerland; SIB Swiss Institute of Bioinformatics, Basel 4056, Switzerland

## Abstract

**Motivation:**

Copy number alterations are driving forces of tumour development and the emergence of intra-tumour heterogeneity. A comprehensive picture of these genomic aberrations is therefore essential for the development of personalised and precise cancer diagnostics and therapies. Single-cell sequencing offers the highest resolution for copy number profiling down to the level of individual cells. Recent high-throughput protocols allow for the processing of hundreds of cells through shallow whole-genome DNA sequencing. The resulting low read-depth data poses substantial statistical and computational challenges to the identification of copy number alterations.

**Results:**

We developed SCICoNE, a statistical model and MCMC algorithm tailored to single-cell copy number profiling from shallow whole-genome DNA sequencing data. SCICoNE reconstructs the history of copy number events in the tumour and uses these evolutionary relationships to identify the copy number profiles of the individual cells. We show the accuracy of this approach in evaluations on simulated data and demonstrate its practicability in applications to two breast cancer samples from different sequencing protocols.

**Availability and implementation:**

SCICoNE is available at https://github.com/cbg-ethz/SCICoNE.

## 1 Introduction

During tumour progression, cancer cells undergo complex and diverse genomic aberrations leading to heterogeneous cell populations and multiple evolving subclones (Greaves and Maley 2012, Nik-Zainal *et al.* 2012, Yates and Campbell 2012). Genomic sequencing has been powerfully used to uncover the heterogeneity across cancer types (Burrell and Swanton 2016) and to examine the link between tumour diversity and progression (Maley *et al.* 2006, Marusyk and Polyak 2010, Merlo *et al.* 2010). Heterogeneity may also allow the tumour additional ways to evolve resistance under treatment, such that intra-tumour genomic diversity is a cause of relapse and treatment failure (Burrell *et al.* 2013, McGranahan and Swanton 2015).

The need for a comprehensive understanding of the composition of each tumour for more precise and effective cancer therapies (Allison and Sledge 2014) can be addressed by sequencing tumours at the resolution of individual cells (Wang and Navin 2015, Gawad *et al.* 2016). The small amount of DNA in single cells has to be amplified before sequencing, which leads to characteristic and pronounced noise in the read count data. Specialized phylogenetic methods for single-cell sequencing data accounting for these noise patterns have been developed to detect point mutations and reconstruct the evolutionary history of tumours (Kuipers *et al.* 2017a; Zafar *et al.* 2018).

In addition to point mutations, cancerous cells often undergo more complex genomic rearrangements, including copy number alterations (CNAs) such as amplifications and deletions. Since the first single-cell DNA sequencing (Navin *et al.* 2011), which allowed for the identification of CNAs, rapid progress has led to high-throughput methods (Zahn *et al.* 2017, Laks *et al.* 2019) that can profile the whole genome of hundreds of single cells. By removing the need for pre-amplification, the protocols of Zahn *et al.* (2017) and Laks *et al.* (2019) have particularly uniform coverage allowing a resolution of CNAs down to the megabase scale. A commercial solution from 10x Genomics was also available for a time, likewise enabling the processing of hundreds of single cells, and used in a large-scale clinical project (Irmisch *et al.* 2021). The resolution and depth of the data depends heavily on the technology used, and the amount of sequencing subsequently performed.

A number of computational tools have been applied or specifically developed for copy number calling in single-cell DNA sequencing data (Garvin *et al.* 2015, Lai *et al.* 2016, Wang *et al.* 2018, Dong *et al.* 2020, Wang *et al.* 2020, Zaccaria and Raphael 2021, Ruohan *et al.* 2022) with reviews and comparisons in overview papers (Mallory *et al.* 2020a,b). The output of these methods is a copy number profile, a partition of the genome into segments, and a sequence of integers indicating the copy number state of each segment. While some of these tools pool information across cells (Wang *et al.* 2020, Zaccaria and Raphael 2021), the tools above do not take into account the shared evolutionary history of cells originating from the same individual or tumour. As previously shown for point mutations, the evolutionary relationships of tumour cells can be used to boost and correct the weak and noisy signal provided by single-cell sequencing reads (Singer *et al.* 2018). However, modelling and inference of evolutionary histories from single-cell data is notably more involved in the case of CNAs, as such events may physically overlap so that treating the events as independent may no longer be appropriate (Mallory *et al.* 2020b).

Methods for jointly calling copy numbers and reconstructing event histories are therefore currently under active development. Previous methods developed for inferring evolutionary histories based on copy numbers assume that copy number profiles of the individual cells are predefined. The methods can be separated into two categories, distance-based approaches (Navin *et al.* 2011, Schwarz *et al.* 2014, Kaufmann *et al.* 2022) and methods that reconstruct the evolutionary history based on a maximum parsimony heuristic (Chowdhury *et al.* 2014, El-Kebir *et al.* 2017, Gertz *et al.* 2016, Wang *et al.* 2021). Further distinction can be made by the way identical copy number changes of neighbouring segments are interpreted, as separate events (McPherson *et al.* 2016), a single event (Schwarz *et al.* 2014, El-Kebir *et al.* 2017, Kaufmann *et al.* 2022), or a hybrid thereof in which deletions/amplifications can either occur separately on individual segments or affect whole chromosomes or genomes (Chowdhury *et al.* 2014, Gertz *et al.* 2016). A limitation shared by all these methods is that errors in the copy number profiles will propagate into the inference of the copy-number trees.

Our approach, SCICoNE (https://github.com/cbg-ethz/SCICoNE), directly integrates the inference of copy number profiles with the reconstruction of copy number event histories tailored to the shallow read-depth of whole-genome single-cell sequencing data. Other tree-based methods have also been developed (Markowska *et al.* 2022, Liu *et al.* 2024): CONET (Markowska *et al.* 2022) builds the tree based on the likelihood of the presence or absence of breakpoints (locations in the genome marking the start or end of a CNA) and calls copy-numbers in post-processing using a discrepancy penalty although this does not directly model the evolution of copy-numbers. NestedBD (Liu *et al.* 2024) instead works in the cell lineage space, treating copy numbers in bins independently, and also calls copy numbers in a later inference step. We therefore benchmark SCICoNE against a suite of eight alternatives, including tree-based methods, and show strong improvements in performance, especially in the low read-depth and high noise regime typical of very shallow whole-genome single-cell sequencing, used for example in Irmisch *et al.* (2021). We further use SCICoNE to examine the copy number history of a xenograft breast cancer sample, and a breast cancer tissue sample processed with the 10x Genomics platform.

## 2 Materials and methods

### 2.1 Model overview

During tumour evolution, CNAs can occur and accumulate in cancer cell subpopulations. Since all cells in a tumour are related through sharing a common ancestor, CNAs occur on a cell lineage tree and encode the copy number profiles of each clone or cell (Supplementary Fig. S1a). For clinical applications, the history of copy number events is often more pertinent than the fully resolved cell genealogy, as the former is sufficient to determine which CNAs are mutually exclusive or co-occurring in the same tumour subclone, and to infer the order of CNA events. We therefore move from the cell or clone lineage tree to the CNA tree (Supplementary Fig. S1b) to model the evolutionary history of the tumour and call copy numbers.

**Figure 1.**
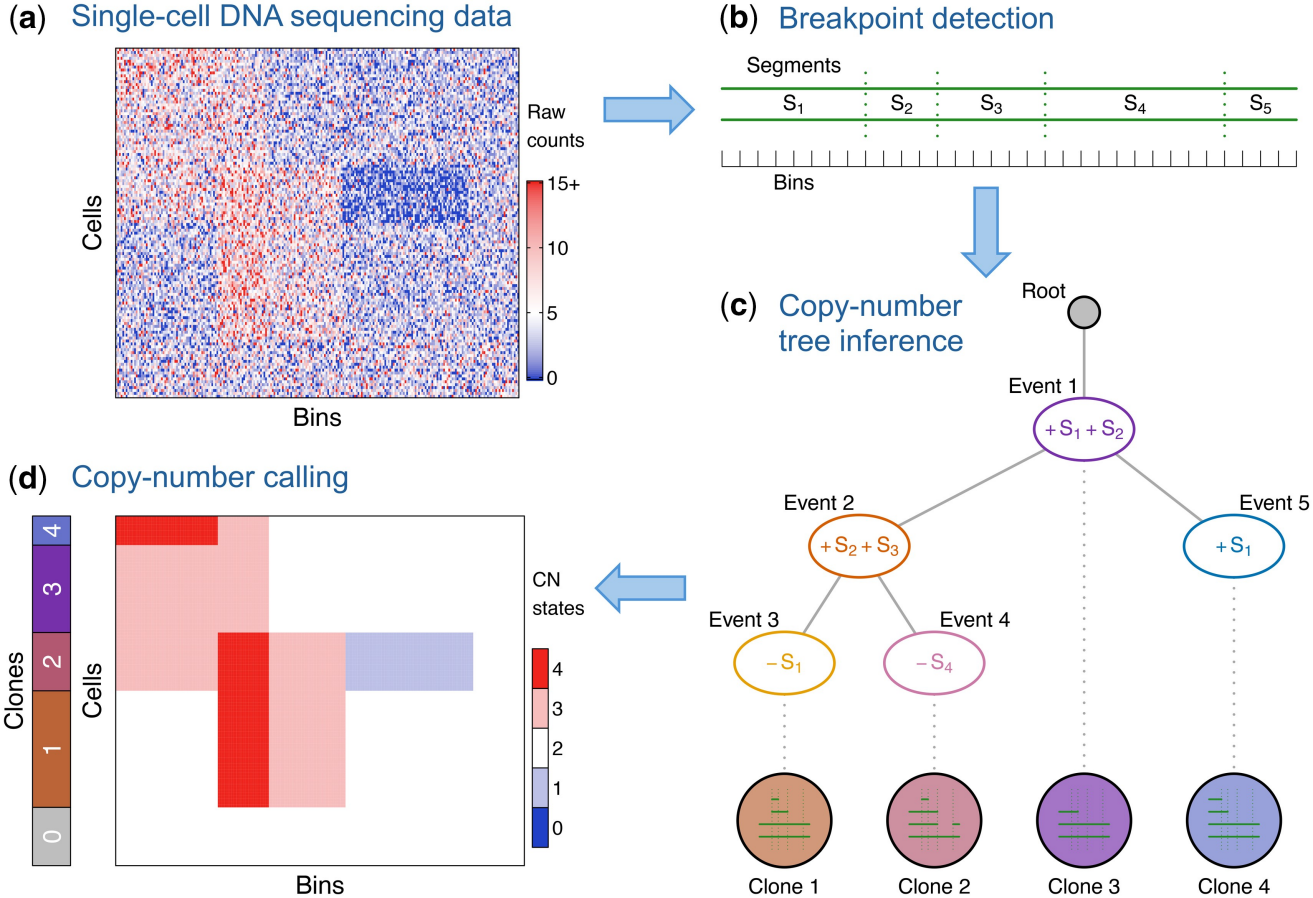
Overview of CNA calling and tree inference with SCICoNE. (a) From single-cell DNA sequencing we obtain noisy read count data reflecting the underlying copy number profiles. (b) As a first step, we detect breakpoints to partition the genome into segments (each comprising several bins) that may experience CNAs. In this case, four breakpoints define the five segments $S_{1},\ldots ,S_{5}$. (c) From the data, we then infer the evolutionary history of copy number changes which accumulate (plus signifies an amplification, minus a deletion) at the nodes of an event tree. By attaching the single-cells to the event tree we obtain their CNAs by tracing the path from the root to call the copy numbers of each clone, or group of cells with the same profile (d). For example, Clone 4 has experienced two CN events after the diploid root, namely Event 1, a gain in a region spanning segments $S_{1}$ and $S_{2}$, and Event 5, a further gain in $S_{1}$, such that the copy number profile of Clone 4 is (4,3,2,2,2) across the five segments.

With the whole-genome sequencing of single cells (10x Genomics, Zahn *et al.* 2017), we have read depth data for each cell and each bin along the genome (Fig. 1a). After correction for confounders, including read mappability and genomic GC content, for each bin (Baslan *et al.* 2012, Garvin *et al.* 2015), the corrected counts can be assumed to be proportional to the underlying copy number. The genome duplication of cell division does not happen everywhere along the genome at the same time so that cells captured during division may have parts of the genome amplified due to the replication rather than reflecting underlying CNAs. To avoid these effects impeding phylogenetic reconstruction, cycling cells should also be filtered (Salehi *et al.* 2023).

To partition the genome into segments of consecutive bins which may have experienced CNAs, we develop a dynamic programming approach to detect breakpoints by combining evidence across the individual cells (Fig. 1b; Section 2.3). For each potential breakpoint, we compare the likelihood of a step change in copy number state to that of a constant model, and then collate the information across cells to obtain the total signal of a copy number change at that genomic position.

To account for the noise in the sequencing data, we develop a probabilistic model and an MCMC inference scheme for single-cell read counts. A major difference to MCMC schemes used for reconstructing trees of point mutations (Jahn *et al.* 2016) is that the infinite sites assumption, which excludes the possibility of multiple mutational hits at the same genomic site, can no longer be made (Kuipers *et al.* 2017b). CNAs may overlap and nest inside each other, so the model developed here allows for arbitrary violations of the infinite sites assumption and arbitrary reoccurrences of amplifications and deletions across different genomic regions. Reoccurrences are only resolved and modelled at the level of the bins of the genomic data, so that the individual break points may still occur at different genomic positions in the same bin. Along with allowing violations of the infinite sites assumption at the bin level, we explicitly model dependencies between bins as they are tied together within copy number events according to the CNA tree model (Fig. 1c).

By jointly estimating the copy number profiles of all cells and their underlying CNA tree, we leverage the evolutionary dependencies among cells for improved copy number calls. The output of SCICoNE comprises both (i) the reconstructed tree representing the evolutionary history of all cells (Fig. 1c) and (ii) the inferred copy number profile for each individual cell (Fig. 1d).

### 2.2 Binning and read count correction

Current protocols for copy number detection at single-cell resolution typically use shallow whole-genome sequencing (≤0.1x coverage per cell) (10x Genomics, Zahn *et al.* 2017) which prohibits coverage-based copy number calling at the level of individual loci. Instead one partitions the reference genome into equal sized bins (20–100 kb) and counts the reads per bin instead of per locus. The raw read count of a bin, is not only determined by the bin’s underlying copy number state, but also by its mappability and GC content. To reduce the bias introduced by these confounders, SCICoNE uses read counts (per bin and per cell) that have been corrected for both effects (Baslan *et al.* 2012, Garvin *et al.* 2015). With these confounders removed, we now assume that the probability of reads falling into each bin is proportional to the bin’s copy number state,

(1) $$p_{i}^{j}\propto c_{i}^{j}$$

where $p_{i}^{j}$ is the probability of a read from cell j falling into bin i, and $c_{i}^{j}$ is the copy number state for that bin in that cell.

### 2.3 Breakpoint detection to define copy number segments

As copy number changes often affect regions much larger than a typical bin size, we collate neighbouring bins into genomic segments with the same copy number state. To collate the bins into genomic segments, we first detect potential breakpoints as bin boundaries where the read depth changes across subsets of cells. For this purpose, we developed a dynamic programming approach that combines evidence across cells to call the breakpoints (Supplementary Appendix A). The detection compares a likelihood-based model of a step change in copy number at each bin to a constant copy number model for each cell and then combines the signal over all cells. Bins with the strongest combined signal relative to a noise threshold are classified as potential breakpoints.

Once the breakpoints have been determined, we collate bins between consecutive breakpoints into segments. For each cell, we sum the counts in all bins belonging to each segment to arrive at a count matrix D with entries $D_{jk}$ for each cell j and each segment $S_{k}$.

The probability of a read falling into segment $S_{k}$ is proportional to the copy number state and the size of the segment

(2) $$p_{k}^{j}\propto c_{k}^{j}s_{k},\;s_{k}=\text{number}\;\text{of}\;\text{bins}\;\text{in}\;\text{segment}\;S_{k}$$

where $p_{k}^{j}$ is the probability of a read from cell j falling into segment k with size $s_{k}$, and $c_{k}^{j}$ is the copy number state for that segment in that cell.

### 2.4 CNA trees

For clinical applications and copy number calling, we work with the history of copy number events represented as a CNA tree (Fig. 1c; Supplementary Fig. S1). This is in analogy to the mutation trees of SCITE (Jahn *et al.* 2016), where the tree nodes now encapsulate events corresponding to amplifications or deletions of the segments. The nodes in the tree can have arbitrary degree. We index the event nodes 1,…,n and additionally label them with the corresponding CNAs. All CNA events are stored in the vector V, such that $V_{i}$ is the collection of CNAs of vertex i. For the example of Fig. 1c, we have $V=(+S_{1}+S_{2},+S_{2}+S_{3},-S_{1},-S_{4},+S_{1})$. The tree structure T we can store as a parent vector T=(0,1,2,2,1) where 0 represents the root.

If cell j is attached to event node $\sigma_{j}$ we can read off the expected copy number state for each segment by tracing all events back to the root for a given tree structure and event vector. In the example of Fig. 1c, the attachment vector is σ=(3,4,1,5). We denote the expected copy number state of cell j for a given attachment point $\sigma_{j}$ as $c_{k}^{j}(T,V,\sigma_{j})$. Then we have for the probability of the reads of cell j falling into segment k
 

(3) $$p_{k}^{j}(T,V,\sigma_{j})=\frac{c_{k}^{j}(T,V,\sigma_{j})s_{k}}{Z^{j}},\;Z^{j}=\sum_{k}c_{k}^{j}(T,V,\sigma_{j})s_{k}$$

If a segment is entirely deleted so that the copy number state and probability is 0, then we would not expect to see any reads in that segment. However, due to mapping errors there may still be some residual reads in that segment. To account for this possibility, we instead set the minimum copy number state to 0<η≪1.

### 2.5 Likelihood

To assess how well a CNA tree with its events, (T,V), fits our read count data, we define a likelihood model as follows. The data matrix entry $D_{jk}$ stores the number of corrected counts cell j has in segment k, with total reads for that cell of $N^{j}=\sum_{k}D_{jk}$. Given the probabilities of reads landing in each segment, we model the data with a Dirichlet-Multinomial distribution to account for overdispersion

(4) $$D_{j}∼\text{Dirichlet-Multinomial}\;(\nu p^{j}(T,V,\sigma_{j})Z^{j},N^{j})$$

where $\nu Z^{j}$ is the concentration parameter (inverse of the overdispersion). We scale by $Z^{j}$ to aid the computational implementation since, using Equation (3), the likelihood contribution from cell j is therefore

$$L_{j}(T,V,\sigma_{j})=\frac{\Gamma \left[\nu Z^{j}\right]}{\Gamma \left[N^{j}+\nu Z^{j}\right]}\prod_{k}\frac{\Gamma \left[D_{jk}+\nu c_{k}^{j}(T,V,\sigma_{j})s_{k}\right]}{\Gamma \left[\nu c_{k}^{j}(T,V,\sigma_{j})s_{k}\right]}$$

In the large ν limit, the model simplifies to the multinomial distribution

(5) $$L_{j}(T,V,\sigma_{j})={\left[Z^{j}\right]}^{-N^{j}}\prod_{k}{\left[c_{k}^{j}(T,V,\sigma_{j})s_{k}\right]}^{D_{jk}}$$

We can compute this likelihood for all cells and all possible attachment points efficiently in total time O(mn) where m is the number of cells and n the number of event nodes. This time complexity is achieved with a tree traversal. During the tree traversal, at each step only the segments in one event node change their state and we only update a limited number of terms in the product. All possible attachment points of a cell can therefore be computed in linear time.

For numerical accuracy, we compute the log-likelihood of all attachment points for each cell relative to the root. When cell j is attached at the root, which is node 0 so that $\sigma_{j}=0$, with a given constant ploidy c, we have the score for that cell of

(6) $$L_{j}(T,V,0)=\frac{\Gamma \left[\nu Z\right]}{\Gamma \left[N^{j}+\nu Z\right]}\prod_{k}\frac{\Gamma \left[D_{jk}+\nu cs_{k}\right]}{\Gamma \left[\nu cs_{k}\right]},\;Z=c\sum_{k}s_{k}$$

whereas for large ν the ploidy constant cancels and we simply have

(7) $$L_{j}(T,V,0)={\left[\sum_{k}s_{k}\right]}^{-N^{j}}\prod_{k}{\left[s_{k}\right]}^{D_{jk}}$$

Since the root is common across all tree models, this contribution only varies if ν is changed and is only recomputed in that case. The root does not need to have constant ploidy; e.g. the sex chromosomes can be set accordingly.

### 2.6 Posterior tree probability

With the likelihoods of each cell for each attachment point, we can directly compute the marginal likelihood when we average over all possible attachment points of each cell in the tree

(8) $$P(D|T,V)=\frac{1}{{\left(n+1\right)}^{m}}\prod_{j=1}^{m}\sum_{\sigma_{j}=0}^{n}L_{j}(T,V,\sigma_{j})$$

with a uniform prior on the attachment points. With a noninformative prior on the tree size, and then a uniform prior also on the trees, the posterior becomes

(9) $$P(T,V|D)\propto \frac{P(V|T)P^{\prime }(T)}{{\left(n+1\right)}^{n-1+m}}\prod_{j=1}^{m}\sum_{\sigma_{j}=0}^{n}L_{j}(T,V,\sigma_{j})$$

where $P^{\prime }(T)$ is a penalization term needed to account for combinatorial effects possible for larger trees which we detail in Supplementary Appendix B. We define and discuss the prior P(V|T) on the event vector in Supplementary Appendix C.

Since we marginalize out the cell attachments, we focus on the CNA tree and build a scheme to find the tree and vector with the highest posterior score. Not all combinations of trees and event vectors are meaningful. After a copy number state of 0 is reached at any point in the tree, the affected segment cannot be regained in any descendant nodes. We then assign a posterior score of 0 to any tree and event vector combination (T,V) where this occurs and set P(T,V|D)=0.

Also, a tree with a placement of events such that it recreates the exact same genotypes repeatedly in different parts of the tree is redundant, as a simpler model using a smaller tree would fit the data equally well. Biologically such convergent evolution may occur, but as it cannot be distinguished by the data itself, we make the assumption of the more parsimonious model. In particular, if we denote $C_{i}(T,V)$ to be the copy number state at node i computed by collating the events in V along the path to the root in the tree T, we forbid the case $C_{i}(T,V)=C_{j}(T,V)$ for any different nodes i and j. To exclude this possibility we assign it a posterior score of 0 and set P(T,V|D)=0 if $\exists i,j|i\neq j\wedge C_{i}(T,V)=C_{j}(T,V)$.

As an alternative to marginalization, we can also place cells at their maximal attachment position and define the score

(10) $$S(T,V|D)=\frac{P(V|T)}{{\left(n+1\right)}^{n-1}}\prod_{j=1}^{m}\underset{\sigma_{j}}{\max}L_{j}(T,V,\sigma_{j})$$

which removes the need for correcting for combinatorial effects via $P^{\prime }(T)$. To distinguish between these two alternatives, we denote inference using the marginalized score, Equation (9), with the term ‘sum’ and inference using the maximized score, Equation (10), with the term ‘max’.

### 2.7 MCMC scheme

To sample trees proportionally to P(T,V|D) we build an MCMC scheme to move in the space of trees and event vectors. We detail the moves in Supplementary Appendix D starting with the simple moves of *prune and reattach* and *label swap* used in SCITE (Jahn *et al.* 2016) which work for a fixed tree size and fixed set of events at the nodes. We additionally weight the move proposals to account for their computational costs. To change the event vector, we introduce an *add or remove events* move, while to change the tree size we developed the *add or remove node* and *condense or split nodes* moves to cover the full discrete space. To aid moving in such a complex space and finding simpler trees which generate the same set of possible genotypes we also introduce a *genotype-preserving prune and reattach* move. Since the set of genotypes is preserved, only the event vector prior needs to be recomputed making this move computationally very efficient, so we score and sample from the whole neighbourhood. For the overdispersion determined by the concentration parameter ν we run a random walk (in log space). For the complete MCMC scheme, at each iteration we first sample a move type with a fixed probability for the six different moves. Each move is equally likely to be chosen apart from the *genotype-preserving prune and reattach* which has a relative weight of 0.4.

### 2.8 Maximal search

We utilize the MCMC scheme to search for the tree with the highest posterior score, keeping track of the maximally scoring tree encountered. From the best scoring tree discovered, we can compute the best attachment of each cell and hence their predicted copy number profiles.

To aid finding the best scoring tree and event vectors, we work stepwise. First we cluster the data [using PhenoGraph (Levine *et al.* 2015)] and construct the average read counts per cluster. The tree likelihood computation only involves running over each cluster (weighted by the cluster size) rather than each cell, giving a large computational speed up. We initialize the states as the normalized read counts rounded to integers, and the tree by performing hierarchical clustering with Ward’s method and filling the events at the internal nodes with our extract common ancestor procedure. We run 10 different chains and check for robustness that at least half the chains return trees with similar high score (the log posterior is within 5 of the maximum). Nonrobust runs are repeated starting from the highest scoring tree found so far. Once the tree on the clustered data has been determined, we run the scheme on the full data, starting from the cluster tree and with the overdispersion learned from the full data with the cluster tree. We compute the distances between the trees returned by half the chains that attain the best scores in the full data, and check for robustness that the average distance is <0.02. We stop the scheme on the full data once either of the robustness criteria are met.

### 2.9 Root state

Instead of assuming a diploid state at the root, it is easy to start with any other state, e.g. tetraploid corresponding to a whole genome duplication. Although the average ploidy is not well defined for read depth data, because CNAs are integer, the differences induced can help determine the underlying states. Under the presence of further CNAs, the model comparison of the tree with a diploid root against that with a tetraploid root potentially allows identification of genome doubling relevant for tumour progression and prognosis (Bielski *et al.* 2018). We identify such a doubling in the 10x Genomics breast cancer dataset.

### 2.10 Simulation settings

For the simulation we consider a genome consisting of 10 000 bins and different numbers of reads per cell with an average of 2, 4, or 8 reads per bin. This is a relatively low read depth per bin, chosen to make the inference more challenging and comparable to the data from the 10x Genomics protocol with their default bin size of 20 kb.

For trees with size n=20, we partitioned the bins into 40 or 80 segments. We sampled the tree structure uniformly and then, at each node in the tree, we sampled the change in copy number by first sampling the number of segments involved with a Poisson distribution with parameter λ=0.1 and added 1, while for each segment we sampled the number of copies with a Poisson distribution with parameter λ=0.2 and again added 1 and sampled the sign uniformly. Trees that violate biological constraints (e.g. having negative copy numbers states) are rejected and resampled. For each setting, we sampled 40 trees.

For each tree, we sample 400 cells, in line with the typical number of cells targeted with the 10x Genomics protocol for a single sequencing run, and sample their attachment point in the tree uniformly. This provides the true copy number profile of each cell, from which the reads per bin were sampled with a Dirichlet-multinomial with concentration ν=4.

For inferring the copy number profiles with our MCMC scheme, we first detected the breakpoints automatically (Supplementary Appendix A with ν=1) to generate the segments. For our MCMC scheme we set $\eta =10^{-4}$ and ran chains of length 4000n, repeatedly until robust results were obtained, to find first the trees on the clustered data and then on the full data. We find the best trees both for the posterior score from the summation of Equation (9) and for the maximization of Equation (10).

For comparison we include HMMcopy (Ha *et al.* 2012, Lai *et al.* 2016) which runs a hidden Markov model on each cell to call the breakpoints and the copy number states which are inferred per bin for each cell individually. For single-cell low-read count data, however, default parameters of HMMcopy should be adjusted and we followed the recommendations of Mallory *et al.* (2020a) (log-transformed counts, $e=1-10^{-15}$, ν=4, strength of $10^{7}$). We also include Ginkgo (Garvin *et al.* 2015), which offers single-cell copy number calling, adapted to deal with cell-by-bin read count matrices and with the baseline, maximum and minimum ploidy set to 2, 6, and 0, respectively, and SCOPE (Wang *et al.* 2020), which we initialize by setting the top 10% cells with lowest Gini coefficient as diploid, and allow at most 20 copy number clones.

We further consider CONET (Markowska *et al.* 2022), a tree-based method where the main difference between SCICoNE and CONET is that CONET does not explicitly model integer copy number changes in their tree nodes, but rather models breakpoint presences or absences as the events in the tree. Copy numbers are called in a post-processing step of mapping to the nearest integer, and while the breakpoints share an evolutionary history, the copy number states are not assumed to, unlike in SCICoNE. The input data for CONET is correspondingly not a cells by regions matrix, but rather the difference in normalized read counts between subsequent bins. For the simulations we followed the recommendations in Markowska *et al.* (2022) and set $k_{0}=1$, $k_{1}=0.01$, s_1_=100 000, s_2_=100 000, and $n_{CN}=2$, and set the number of parallel tempering iterations to 200 000. In case the method took >24 h to run, we reran with their default setting of 100 000 iterations. CONET also requires candidate breakpoints as input for which we used the scheme provided by their software which relies on CBS (Olshen *et al.* 2004) and MergeLevels (Willenbrock and Fridlyand 2005) for genome segmentation.

We also include NestedBD (Liu *et al.* 2024), which takes as input the single-cell copy number profiles learned by Ginkgo and finds a lineage tree relating them. To enable inference on the 400 simulated single-cell profiles, we first apply hierarchical clustering on the Ginkgo copy number profiles to obtain 20 copy number clusters. After the NestedBD tree inference on the cluster medians, we use the corrected copy numbers per cluster provided by the method to obtain the single-cell copy number profiles, and compare with the ground truth. We used 100 000 MCMC iterations to fit our computational budget and discarded the default first 20% as burn-in. We obtained the maximum clade credibility tree using BEAST (Bouckaert *et al.* 2014) and extracted the variance for the normal error model by taking the mean of the posterior samples of the parameter ‘VR’ squared. The NestedBD code additionally required an ‘orig_dist’ variable which we set as the posterior mean of ‘OrigTime’ from BEAST.

We additionally include a diploid profile and two clustering methods. For the clustering we use both PhenoGraph (Levine *et al.* 2015) and hierarchical clustering (with the number of clusters chosen according to the Calinski-Harabasz index) and assigned all cells in each cluster the average read counts of that cluster. For each cluster and its associated cells, we then set the median counts per cell to 2 (by doubling and dividing by the median) assuming that diploid is the most common state and rounded the copy number states to the nearest integer. In the comparison, we compute Δ as the root mean squared difference over all cells and bins, between the true copy number profiles and the inferred values.

For the comparison, we used the distance Δ between the inferred and simulated copy number states, rather than a distance between the structures of the inferred and simulated trees since none of the alternative methods generates a CNA tree. The distance Δ works on the set of predicted genotypes, so that errors in the tree structure which generate incorrect genotypes for cells will be picked up by this distance measure.

As a further measure we adapt the path difference metric (Steel and Penny 1993), previously used for clone and mutation trees (Yuan *et al.* 2015, Jahn *et al.* 2016), to CNA trees. Specifically we define a distance between a pair of cells on tree T along the shortest path between the cells through their most recent common ancestor. Along the path we count the total number of events encountered, with each event weighted by the number of bins it affects. Equivalently for each bin we count how many times it changes state, and by how much, along the path and sum over all bins:

(11) $$d_{ij}(T)=\frac{n_{s}}{n_{b}}\sum_{b=1}^{n_{b}}\sum_{\begin{array}{c} e\in \gamma_{T}\\ \left(i\to j\right) \end{array}}\left|\text{change}\;\text{in}\;\text{state}\;\text{along}\;\text{edge}\;e\;\text{for}\;\text{bin}\;b\right|$$

where $n_{b}$ is the number of bins, $n_{s}$ the number of segments and $\gamma_{T}(i\to j)$ the shortest path between cells i and j on tree T. We normalize the distance to count in units of ‘typical’ events by scaling by the expected size of an event ($\frac{n_{b}}{n_{s}}$). To compare an inferred tree $T^{\prime }$ to the true generating tree T we compute the root mean square difference between the (triangular) distance matrices for the two trees

(12) $$\tau =\sqrt{\frac{2}{m(m-1)}\sum_{\begin{array}{c} i,j\\ i<j \end{array}}{\left(d_{ij}(T)-d_{ij}(T^{\prime })\right)}^{2}}$$

When log-transforming τ for plots, to avoid numerical issues we add an offset of $\sqrt{\frac{2}{m(m-1)}}$ corresponding to a difference of a single event between one pair of cells.

### 2.11 Convergence analysis

Since searching and sampling in the space of event trees can be challenging, we examine the convergence of SCICoNE by running multiple chains and comparing their outputs. Across 10 chains we retain the half with the highest posterior or maximized likelihood score and compare their similarity with two metrics: (i) the average pairwise tree distances τ among the five trees, and (ii) their average difference in log score from the best tree. We ran several rounds of shorter chains (10 000 iterations) for the simulation setting of 20 nodes and 4 reads per bin. As the base case, we generated the data from uniformly sampled trees and started the tree inference from the tree learned from the clustered data. This process is repeated 40 times. The separate runs converge to similar solutions after several rounds (Supplementary Fig. S8, middle column), both in terms of tree distances and their scores.

If we generate data from linear trees instead of uniformly sampled random trees, the convergence is very similar, though there are a few simulations that require some additional rounds (Supplementary Fig. S8, left column). If we start the inference from a random tree instead of using the result from the clustered data, we observe very similar convergence, though with slightly larger score differences in the early rounds (Supplementary Fig. S8, right column). These observations suggest the scheme is robust to different types of evolutionary trees and the initialization of the inference. Finally, we also considered smaller trees with 10 nodes, where convergence is notably faster (Supplementary Fig. S8, bottom row), as would be expected given the smaller search space for trees with fewer nodes.

## 3 Results

### 3.1 Reconstructing copy number trees from tumour data

As a first demonstration of SCICoNE on whole-genome single-cell sequencing data, we considered a dataset of 260 cells of a triple negative breast cancer xenografted into a mouse (SA501X3F) (Zahn *et al.* 2017). The read-count data consists of 18 175 bins of 150 kb size, with a mean coverage of 153 reads per bin per cell (standard deviation of 82). The segmentation algorithm of SCICoNE (Section 2.3, with default parameters) revealed 316 candidate breakpoints.

**Figure 2.**
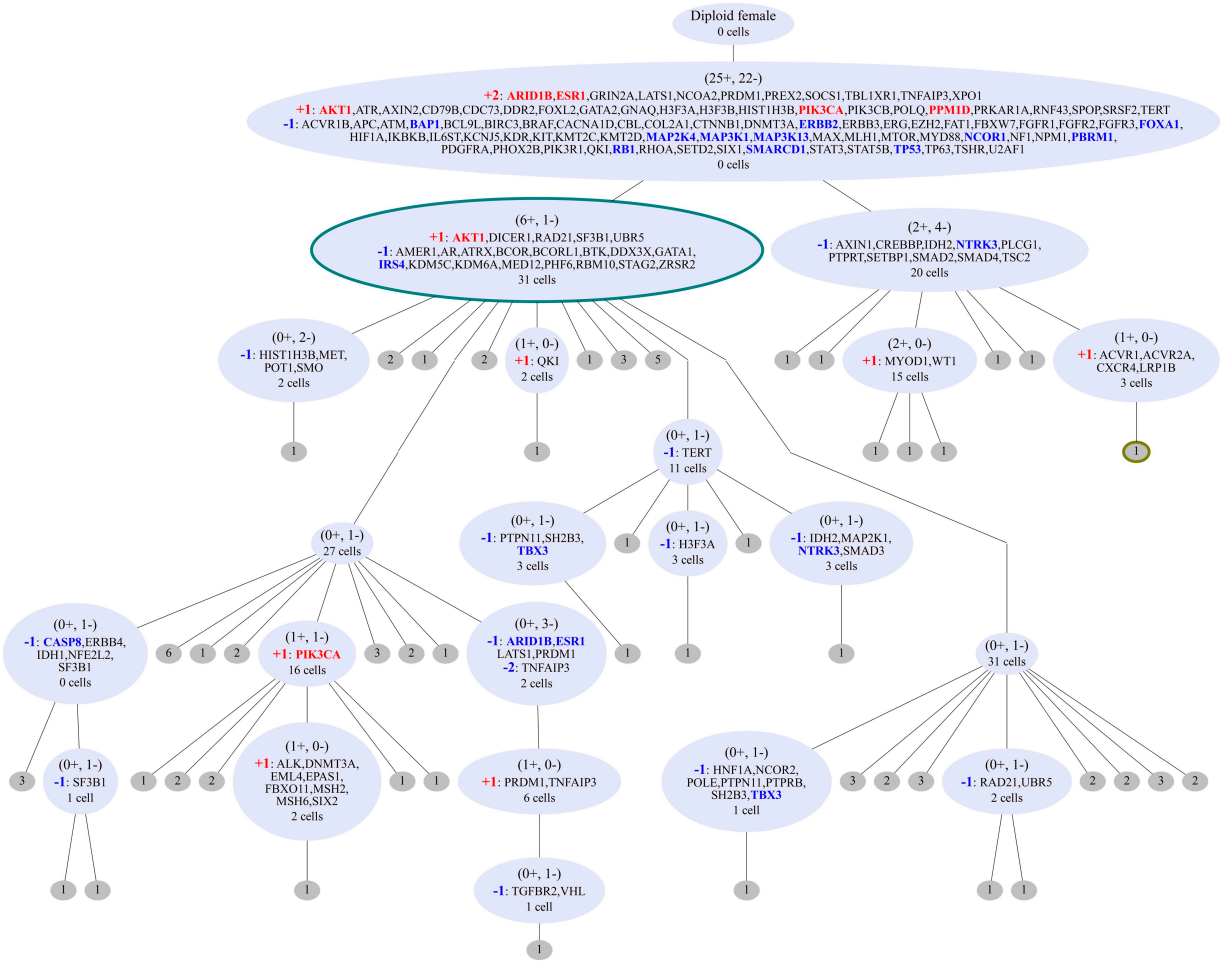
Inferred tree for 260 cells from a breast xenograft (Zahn *et al.* 2017). Inside the nodes of the CNA tree we highlight the total number of amplification or deletion events (in parentheses), the genes which are affected [amongst all genes from the COSMIC Cancer Gene Census (Sondka *et al.* 2018), with the 33 associated with breast cancer highlighted], including how much they are amplified or deleted, and the number of cells that best attach to each node. The CNAs are not displayed at the grey (leaf) nodes where only the number of cells attached is indicated. The number of cells attached to the leaf and internal nodes combine to the 260 cells in total. Example profiles of cells attaching to the two nodes with coloured (thick) borders (one from the largest subclone and one from the other main lineage) are displayed in Fig. 3c.

The reconstructed tree (Fig. 2) starts with a large number of CNAs and then branches into two main populations which further differentiate into different clones. The CNA events are learned at a segment, and hence bin, level onto which we then map genes through their genomic coordinates to describe CNAs at the gene-level. The first node includes deletion in *TP53*, which is a common early driver of triple negative breast cancer (Koboldt *et al.* 2012). The first node also includes deletions in the *MAPK* genes (which are on different chromosomes) along with an amplification in *PIK3CA* and *ATK1*. Aberrations of the MAPK pathway have been linked to tumourigenesis, for which crosstalk with an activated PI3K/AKT pathway has been further implicated (Hashimoto *et al.* 2014), aligning with the copy number state of the ancestor node in the tree. Notably, *AKT1* undergoes repeated amplifications, while *ARID1B* and *ESR1* (both on chromosome arm 6q) are first amplified at the root and later undergo a deletion in one sub-branch. The genes *NTRK3* and *TBX3* instead experience deletions each in two parallel lineages. This may indicate chromosomal instability leading to repeated CNAs and the importance of allowing such recurrence in the evolutionary modelling. Overall, the tree highlights the complex evolutionary history of the sample.

**Figure 3.**
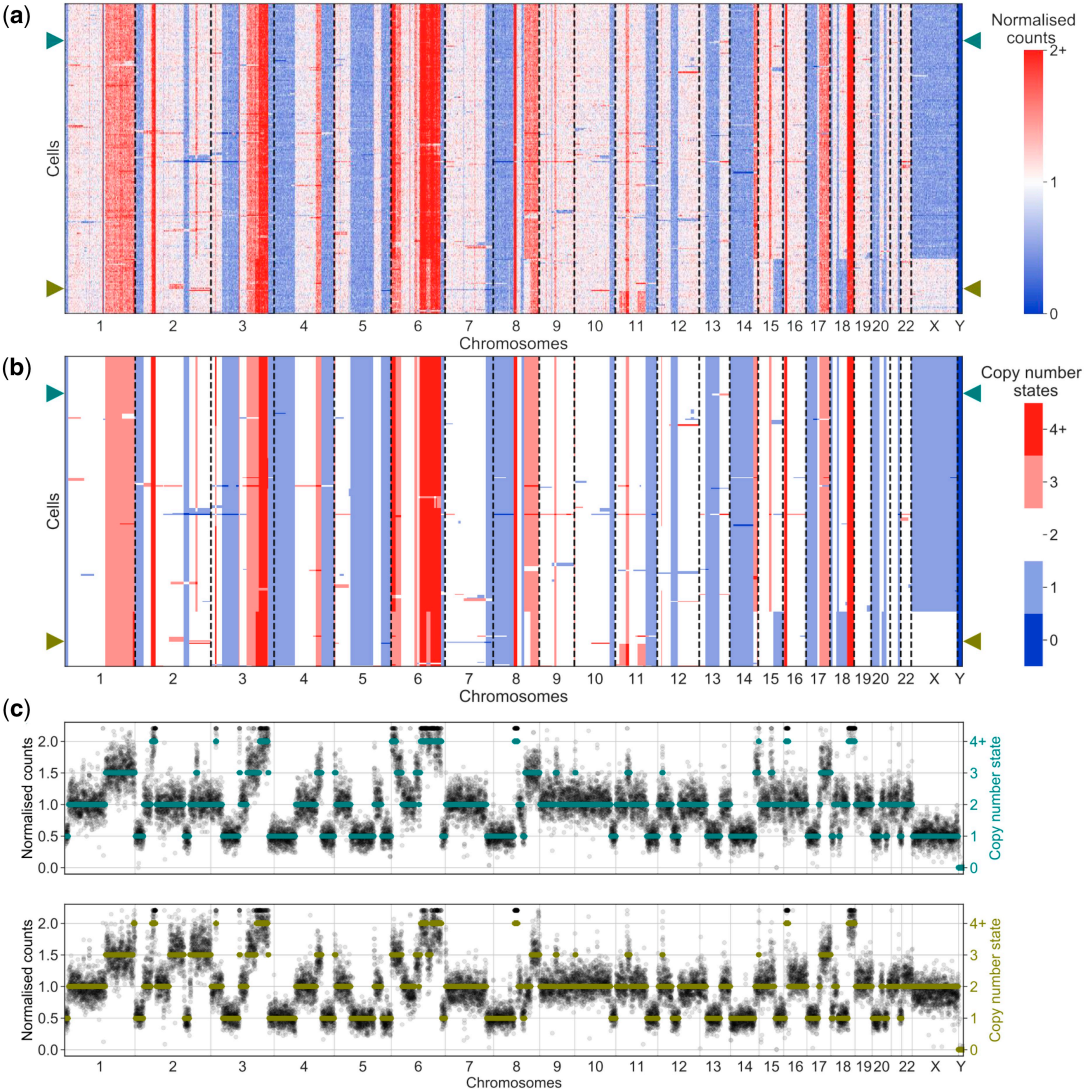
Inferred copy number profiles for 260 cells from a breast xenograft (Zahn *et al.* 2017). (a) Normalized counts per bin, ordered according to the tree in Fig. 2. (b) Copy number profiles estimated jointly with the CNA tree of Fig. 2. (c) Two examples of raw count data (black dots) and inferred copy number profiles (coloured lines) of the two cells indicated by arrows in the heatmaps.

The inferred copy number profiles (Fig. 3b) are in good agreement with the normalized counts per bin (Fig. 3a), recapitulating the CNAs across cells while accounting for the noise in the raw data and the phylogenetic relationships between the cells. Joint inference of copy number profiles and their evolutionary history provides increased power to separate signal from noise, as emphasized by comparing the raw count and inferred copy number profiles of example cells (Fig. 3c). While some small changes for individual cells visible in the normalized counts (Fig. 3a) are filtered out, most changes occurring even in a small number of cells are detected (Fig. 3b). Overall, we obtain the main CNAs across cells as well as their phylogeny (Fig. 2).

To examine the relationship between CNAs and RNA expression changes at the single-cell level, we analysed the xenograft passage SA501X2B which underwent single-cell RNA sequencing (Campbell *et al.* 2019) and compared the two molecular profiles. By smoothing the RNA signal (Tirosh *et al.* 2016), we observe common strips of over- and underexpression (Supplementary Fig. S2). Comparing to the DNA summarized at the gene level (by averaging over the bins in each of the 6000 expressed genes), we find some agreement between genomic copy number changes and expression levels (Supplementary Fig. S2). However, quite a few of the signals visible in the RNA data, like in chromosome 19, e.g. have no basis in the DNA. The concordance and discrepancies between RNA expression and DNA count levels is further emphasized if we cluster the cells (Supplementary Fig. S3). The finer structure and clear breakpoints visible in the DNA, which are reflected in the inferred evolutionary history (Fig. 2) and copy number profiles (Fig. 3) are not well reflected in the RNA (Supplementary Fig. S2). Moreover, the correlation between RNA expression profiles and DNA read counts, while present, is fairly weak (Supplementary Fig. S4). The results confirm on the level of individual cells that expression profiles are not in a strict one-to-one correspondence with copy number profiles and that the latter can be inferred with higher accuracy and in more detail from DNA data.

**Figure 4.**
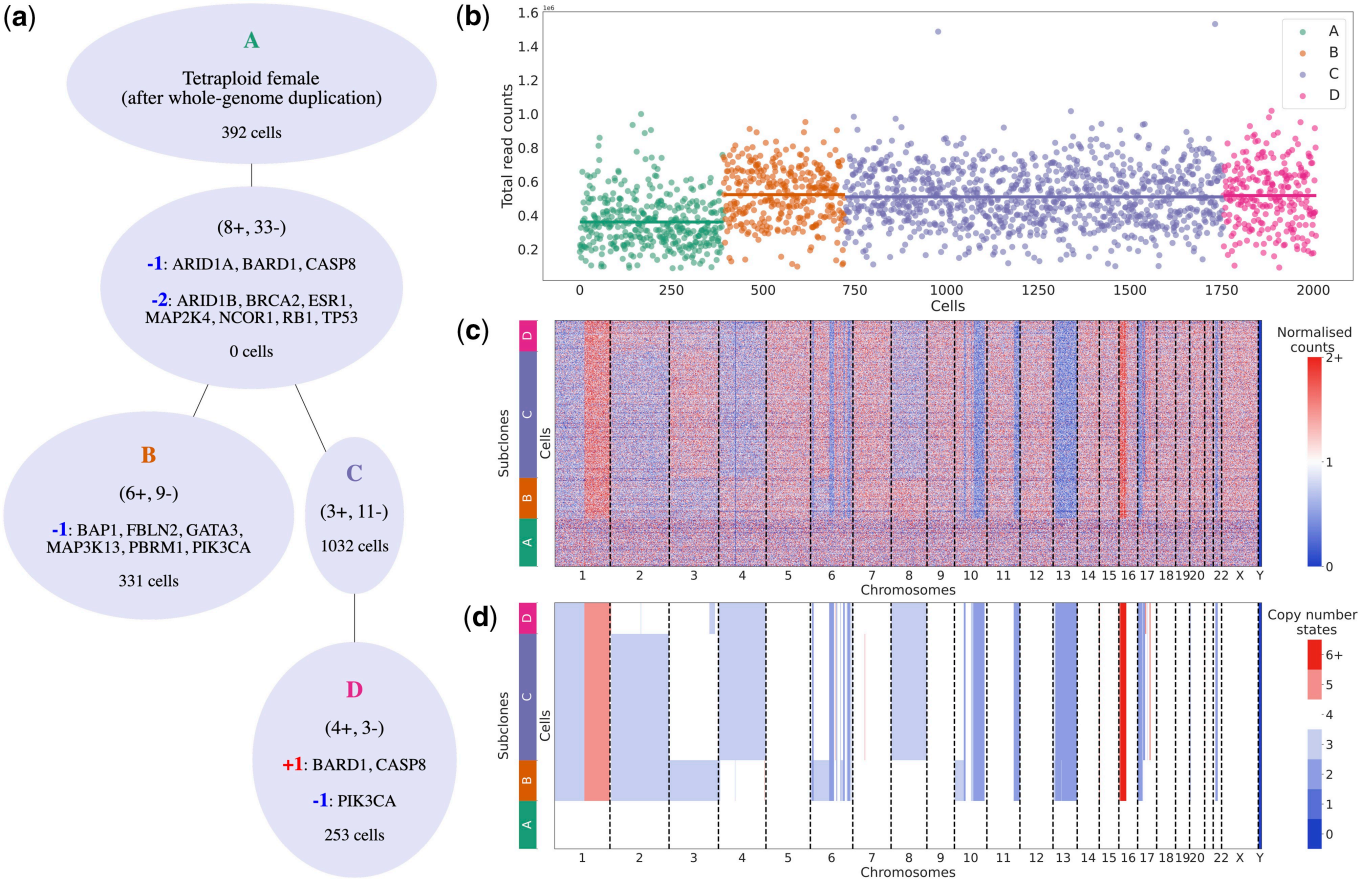
Inferred copy number profiles for 2053 cells from a breast cancer (10x Genomics). (a) Inferred tree on the clustered data, with the genes which are affected [among the 33 associated with breast cancer in the COSMIC Cancer Gene Census Sondka *et al.* 2018] displayed at each node. (b) Counts per cell for the different clusters. (c) Normalized counts per bin. (d) Copy number profiles estimated jointly with the CNA tree.

Next, we examined 2053 single-cells from a triple negative breast cancer available as an example dataset from 10x Genomics (Breast Tissue nuclei section E). The 10x Genomics Cellranger pipeline filtered out 45 cells as low quality and performed GC correction, while we removed 57 outlier bins with >3 times the median counts. To robustly detect breakpoints from the 20 kb-sized bins, we used a window size of 100 bins, allowing for the detection of copy number events that span at least 2 Mb. Already from the read counts per cell (Fig. 4b), we can see a difference between the clusters based on the read-count profiles (Fig. 4c), with higher levels in the tumour cells (clones B–D) pointing to a possible whole-genome duplication. Comparing the results of SCICoNE with a diploid root against a run with a tetraploid root including a genome duplication, we observe much higher likelihoods in the latter case. We therefore proceed with a tetraploid root, for which the resulting cluster tree and copy number calls (Fig. 4a and d) recapitulate the main clonal architecture and their evolutionary relationships. Though the tree inference is run with a tetraploid root, cells that attach to the root do not display CNAs (Fig. 4, clone A) and can be reassigned as diploid nonmalignant cells in post-processing. The whole-genome duplication then becomes the initial event of the tumour clones.

### 3.2 Benchmarking on simulated data

**Figure 5.**
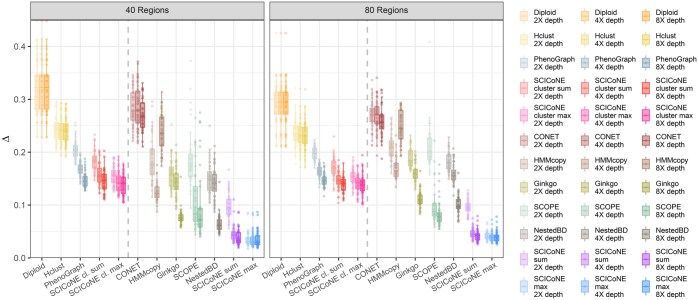
Comparison of copy number calling for simulated data. For uniform random trees with 20 nodes, we attached 400 cells and simulated overdispersed read data according to each cell’s copy number profile over 10 000 bins. The total number of reads was 20k, 40k, and 80k for an average read depth of 2X, 4X, and 8X (colour intensity, left to right) per bin for each cell. The maximal number of segments affected by copy number changes was 40 and 80 (panels). The root mean squared difference Δ between the true simulated copy number profiles and the corresponding inferred profiles over all bins and cells is summarized in each box plot (generated with ggplot2 default settings), for a neutral diploid profile, for profiles inferred by hierarchical , and PhenoGraph clustering as well as SCICoNE on PhenoGraph clustered data, followed by CONET (Markowska *et al.* 2022), and HMMcopy (Lai *et al.* 2016), Ginkgo (Garvin *et al.* 2015), and SCOPE (Wang *et al.* 2020), NestedBD (Liu *et al.* 2024), and SCICoNE on the full single-cell sequencing data. The comparison with a logarithmic transform of Δ is displayed in Supplementary Fig. S5.

To benchmark SCICoNE we conducted a simulation study (Section 2.10), mimicking the very shallow coverage of a recent large clinical project (Irmisch *et al.* 2021), and compared the performance of SCICoNE to a suite of alternatives. For simulated trees with 20 nodes (Fig. 5 and Supplementary Fig. S5), we observe a steady increase in accuracy as we model our data in more detail. We first consider methods that cluster the data, and as a baseline, we cluster the normalized count data using hierarchical clustering or PhenoGraph (Levine *et al.* 2015) (Fig. 5) and assign cells the averaged profile of their clusters (rounded to integers and centred at diploid). Then we build trees on PhenoGraph clustered data using SCICoNE to leverage information across the clusters and their shared evolutionary history to improve the accuracy of learning the copy number profiles (Fig. 5). Next, we compare to methods that work at the single-cell level, and we perform the full tree inference with SCICoNE (Fig. 5), observing a strong improvement over the alternatives. On the full data, we find that the ‘max’ setting, which finds the best placement of each cell in the CNA tree, offers higher reconstruction quality relative to ‘sum’, which averages over their placements [Section 2, Equations (9) and (10)]. We therefore used ‘max’ for the breast cancer data above.

SCICoNE performs well in comparison to HMMcopy (Lai *et al.* 2016) (Fig. 5, bronze) which performs copy number calling per cell with a hidden Markov model, and has been found to have good overall performance in single-cell copy number calling (Mallory *et al.* 2020a). However, for the simulated data with an average read depth of 2–8 (comparable to 10x Genomics data), HMMcopy can have quite variable performance and is generally worse than the Phenograph clustering (Fig. 5, silver). Instead we found Ginkgo (Garvin *et al.* 2015) (Fig. 5, olive) to perform similarly to PhenoGraph clustering at lower depths and with more segments (and smaller copy number events) but better with fewer segments and especially at the highest depth. NestedBD (Liu *et al.* 2024) aims to improve the output of Ginkgo by enforcing that the cells are organized in a lineage tree, and we accordingly observe a slight improvement. SCOPE (Wang *et al.* 2020) (Fig. 5, green) is better still at the higher depths and a little worse than Ginkgo at the lowest depth, although its performance is quite variable across the repetitions. In contrast, SCICoNE on the clustered data is very robust to the read depth and performs better than the other clustering approaches (hierarchical and PhenoGraph), while on the full (un-clustered) data SCICoNE allows us to extract much more accurate copy number profiles than alternative (HMMcopy, Ginkgo and SCOPE) methods, along with the evolutionary history encoded in the tree itself. We observe similar performances in the easier setting of higher coverage [more akin to the coverage of the protocols of Zahn *et al.* (2017) and Laks *et al.* (2019)] and no overdispersion (Supplementary Fig. S6). This leads to a clearer improvement of NestedBD over Ginkgo and SCOPE, while SCICoNE still offers the best performance. Also HMMcopy performs notably worse due to it often calling the reference level incorrectly, while using SCICoNE to build a tree on the clustered data does not offer an advantage in copy number calling compared to the clustering itself.

In terms of runtimes (Supplementary Fig. S7), methods that do not learn a tree and which cluster the data (Hclust and PhenoGraph) are very fast, followed by those which call copy numbers per cell (HMMcopy and Ginkgo), apart from SCOPE which is quite computationally intensive. Reconstructing a tree is however typically more than an order of magnitude more demanding in terms of runtime and the various tree inference algorithms have a similar order of magnitude to each other (Supplementary Fig. S7, right panel). Although the exact runtime may depend on factors like the parameter settings and how breakpoints are detected (Section 2), SCICoNE is among those with longer runtimes, though with the benefit of learning better trees and calling the copy numbers more accurately.

**Figure 6.**
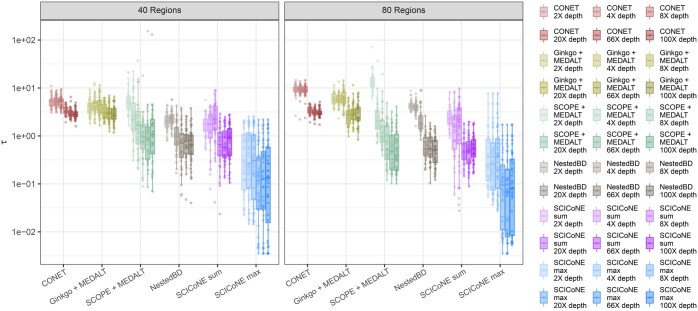
Comparison of copy number tree reconstruction for simulated data. For the simulated data of Fig. 5 and Supplementary Fig. S6 we compute the tree distance τ (Section 2.10) between the true and inferred tree from CONET (Markowska *et al.* 2022), MEDALT (Wang *et al.* 2021) run on the output of Ginkgo (Garvin *et al.* 2015), and SCOPE (Wang *et al.* 2020), NestedBD (Liu *et al.* 2024) as well as SCICoNE. Since the tree distances cover several orders of magnitude we use a logarithmic axis.

CONET (Markowska *et al.* 2022), despite using a phylogenetic tree, performs very poorly in the simulations, at odds with the results they report. We tracked this discrepancy down to their simulations implicitly providing CONET with effectively much higher read-depth data than they provided to SCICoNE, and perform a detailed analysis in Supplementary Appendix E. To benchmark the tree learning we take the output of Ginkgo and SCOPE as input for MEDALT (Wang *et al.* 2021) which reconstructs a phylogenetic tree from called copy numbers, and compare to the trees from CONET (Markowska *et al.* 2022), NestedBD (Liu *et al.* 2024), and SCICoNE (Fig. 6). Similarly to the copy number calling (Fig. 5), CONET is the worst performer, but close to the performance of Ginkgo with MEDALT. SCOPE offers a better input to MEDALT than Ginkgo at the higher depths, but worse at the lowest, with quite high variability as a function of depth. NestedBD clearly improves over Ginkgo with MEDALT, especially at the higher coverages where it is comparable to SCOPE with MEDALT and the ‘sum’ setting of SCICoNE. Overall, the best performance comes from modelling the data with SCICoNE with the ‘max’ setting, in line with the results for copy number calling.

## 4 Conclusions

For learning the copy number profiles of single cells, sharing information across cells and leveraging their shared evolutionary history boosts our ability to remove noise and accurately call copy numbers. Here we developed a novel phylogenetic framework for this purpose, enabling us to jointly infer the tree and copy number states and obtain better quality profiles. When learning the sequence of CNAs that occur in tumour samples from single-cell sequencing data, the possible overlap and reoccurrences of CNAs need to be accounted for. Our tree model allows regions of the genome to be arbitrarily amplified and deleted, while controlling for model complexity. Simulations demonstrate that our approach is accurate for the challenging task of reconstructing the evolutionary history of tumours and calling copy numbers in individual cells.

For a real data example of 260 xenograft breast cancer cells, we obtained the CNA tree and inferred copy number profiles of the cells and detected the main clonal structure as well as their phylogenetic relationship. Some smaller or weaker changes over small numbers of cells, visible in the normalized data (Fig. 3a) were not detected as copy number changes in the inference (Fig. 3b) as they were insufficient to justify a more complex model in our framework. Adjusting and relaxing the penalization for model complexity to detect finer changes may offer further improvements, though learning larger and more complex trees also increases the computation cost of the inference.

For the 2053 cells from a triple negative breast ductal carcinoma, sequenced with the 10x Genomics technology, we could detect a whole-genome amplification and use this to correct the ploidy for the tumour cells to accurately call their copy number states.

To learn the segments in the first place, we developed a dynamic programming approach to combine the evidence of breakpoints across all cells. Compared to methods which work on a per cell basis, our joint inference of breakpoints and then the full probabilistic model of the phylogeny provides a substantial improvement. The combination of bins into segments reduces the possible search space and speeds up the inference, but since the quality of the segments detected directly affects the downstream reconstruction, further improvements in this direction are important. In particular, once a phylogeny has been learnt based on the strongest breakpoints, the corresponding separation of cells into clones can help distinguish noise from signal in the breakpoint detection. Adding and removing breakpoints could also be incorporated as a move into the MCMC scheme itself.

For real data, where GC and mappability corrections are performed to normalize the bins, residual confounding effects still remain which can complicate the breakpoint detection and phylogenetic inference. In particular, the bin corrections depend on the underlying copy number state, indicating that future directions could consider jointly inferring the corrections along with the segments and the phylogeny, although increasing the complexity and computational cost of learning CNA trees.

Understanding and reconstructing the history of copy number events in a tumour could play a key role in predicting response to treatment, especially when resistance arises from adaptive selection of the existing clonal architecture. The combination with single-cell transcriptomics can uncover the interplay between evolutionary pressure and cellular reprogramming (Kim *et al.* 2018). The phylogenetic methods developed here for large-scale, complex and overlapping events could potentially also reconstruct trajectory structures reflected in the transcriptomic profiles of single-cell RNA sequencing, while the copy number trees reflect a common structure on which to analyses expression profiles (Campbell *et al.* 2019, Ferreira *et al.* 2021).

Although developed for shallow whole-genome single-cell sequencing protocols (10x Genomics, Zahn *et al.* 2017, Laks *et al.* 2019, Irmisch *et al.* 2021), our phylogenetic methods may also play a key role in copy number reconstruction for targeted single-cell sequencing (MissionBio), and offer relevant methodology for phylogenetics which integrate copy numbers and point mutations (Sollier *et al.* 2023) and for considering allele-specific events where methods currently look at the phasing through mutations but without jointly learning the phylogenetic history (Zaccaria and Raphael 2021, Ivanovic and El-Kebir 2024). Copy number reconstruction further complements multi-faceted single-cell profiling (Irmisch *et al.* 2021), e.g. to determine the downstream effects of tumour heterogeneity through evolutionary analyses across cohorts. Accurate copy number calling at the single-cell level, enabled through SCICoNE’s joint inference of the evolutionary history and copy number profiles, will enhance single-cell analysis for cancer biology.

## Supplementary Material

btaf072_Supplementary_Data

## Data Availability

The xenograft sequencing datasets utilized in this study were generated by Zahn *et al.* (2017) and Campbell *et al.* (2019) and are available at the European Genome-phenome Archive (http://www.ebi.ac.uk/ega/) under accession number EGAS00001002170 and EGAD00001004552. The dataset from 10x Genomics can be downloaded from https://support.10xgenomics.com/single-cell-dna/datasets as ‘Breast Tissue nuclei section E 2000 cells’.

## Code availability

SCICoNE has been implemented in C++ and is freely available under a GNU General Public License v3.0 license at https://github.com/cbg-ethz/SCICoNE. The code repository also provides a Python package to embed SCICoNE with data pre-processing steps and downstream analyses of the results, and includes an interface for data generated with CellRanger (10x Genomics). We additionally provide Snakemake (Mölder *et al.* 2021) workflows to reproduce all our results.
