# Supplementary material for: Single-cell copy number calling and event history reconstruction
Source: Bioinformatics. 2025 Feb 13;41(3):btaf072. doi: 10.1093/bioinformatics/btaf072 (PMC11897432; doi:10.1093/bioinformatics/btaf072)
Supplement: btaf072_Supplementary_Data [file btaf072_supplementary_data.pdf]

Supplementary Material:

Single-cell copy number calling and event history reconstruction

Jack Kuipers[1,2]*, Mustafa Anıl Tuncel[1]*, Pedro F. Ferreira[1,2]*, Katharina Jahn[1,2] and Niko Beerenwinkel[1,2]

[1] *Department of Biosystems Science and Engineering, ETH Zurich, Basel, Switzerland*
[2] *SIB Swiss Institute of Bioinformatics, Basel, Switzerland*

Supplementary Figures

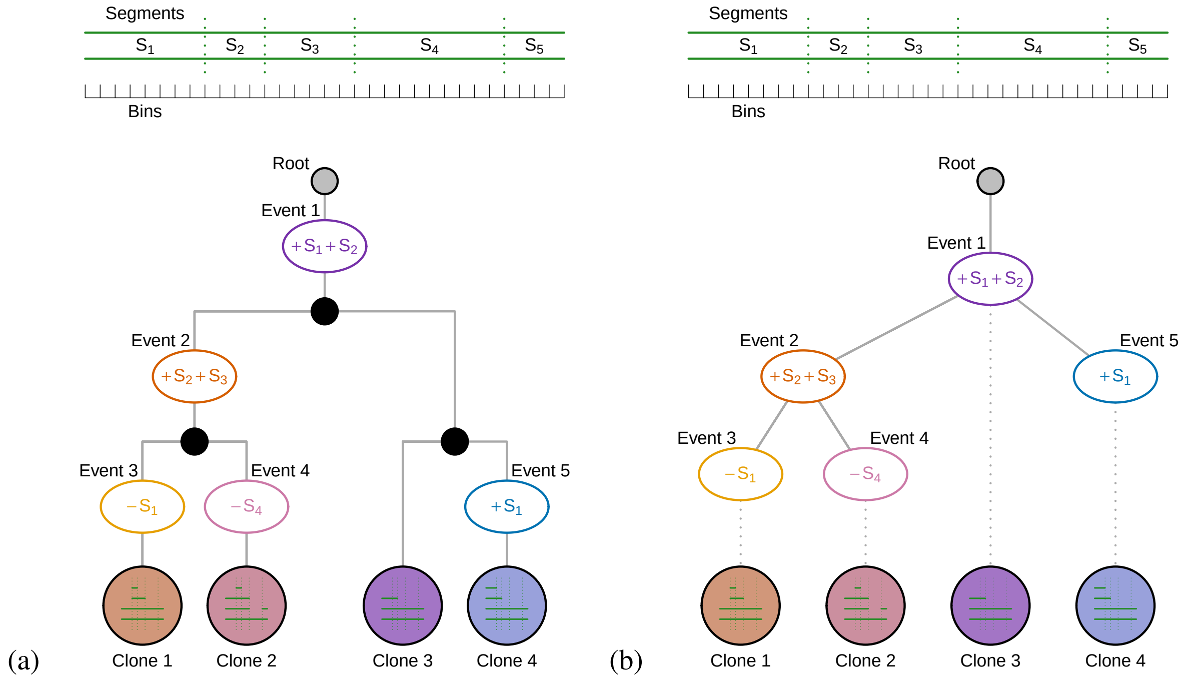

Figure S1: **Clone lineage and CNA trees.** (a) In a cell lineage representation, CNAs affecting segments of the genome (top) accumulate along the branches of a binary tree with splits when clone lineages start to differ. Alternatively we can represent the same set of possible copy number profiles in a CNA tree (b). In this representation the events become the nodes of a tree, with clones attached to event nodes. In both representations the copy number profiles of clones is obtained as the accumulation of all events along the path from the root.

*Contributed equally

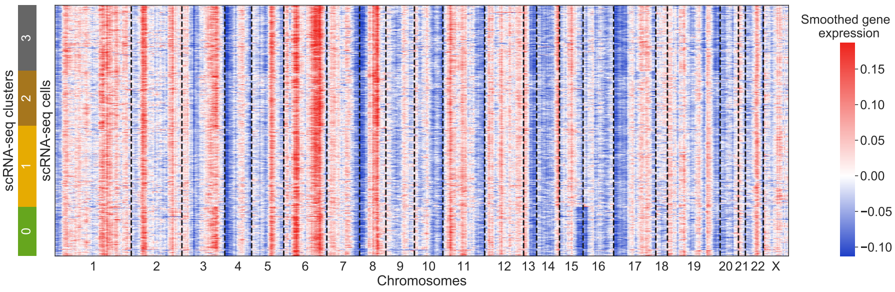

(a)

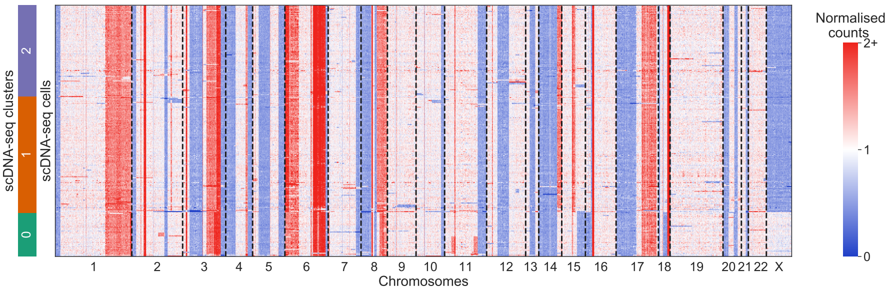

(b)

Figure S2: **RNA and DNA levels for breast xenograft cells.** (a) The smoothed expression profiles of 1152 cells from single-cell RNA sequencing [1]. (b) The normalised counts per gene for the expressed genes for 260 cells from single-cell DNA sequencing [2] are displayed for comparison.

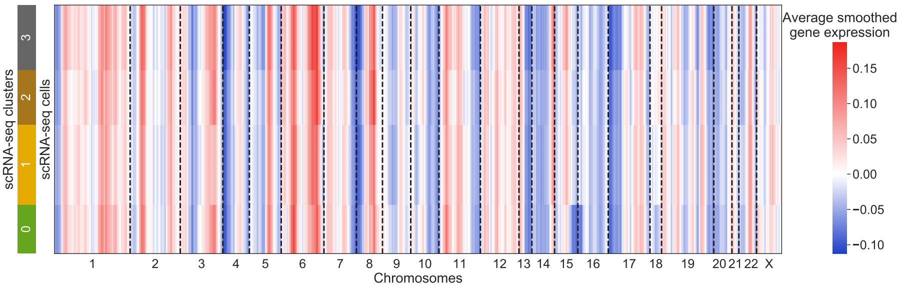

(a)

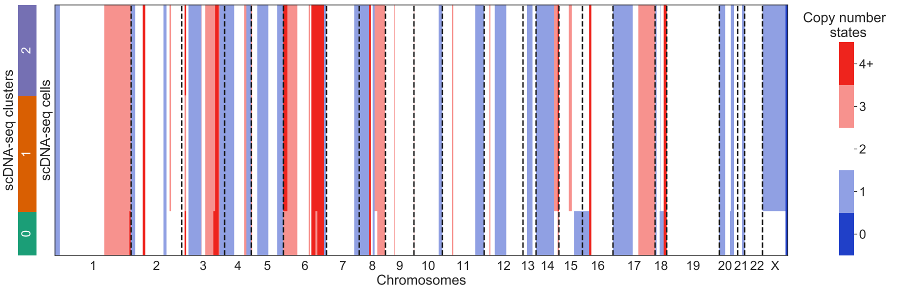

(b)

Figure S3: **RNA level and DNA copy number for breast xenograft clusters.** (a) The smoothed expression profiles of the cells in Figure S2a are clustered and the average profile of the cluster displayed. (b) The inferred copy number profiles of clones built from clustering the single-cell DNA sequencing data and learning a tree using SCICoNE (displayed at the gene level to match the normalised counts in Figure S2b). The inferred copy number profiles of the full data are in Figure 3.

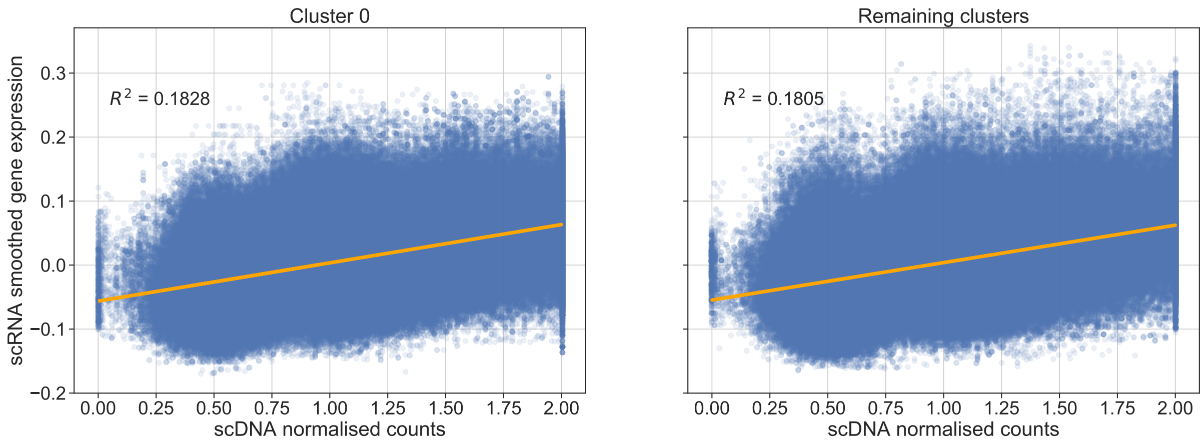

Figure S4: **Correlation between RNA and DNA profiles.** For the smoothed RNA expression and normalised DNA count profiles (Figure S2), we look at the correlation between the two modalities. Since different cells underwent each sequencing, we compare an RNA cell to a DNA cell both from cluster 0 (left panel) or both from other clusters (right panel) and plot the RNA expression level and DNA count depth for each gene. This is repeated for 100 random pairs of cells to obtain each scatter plot and correlation line.

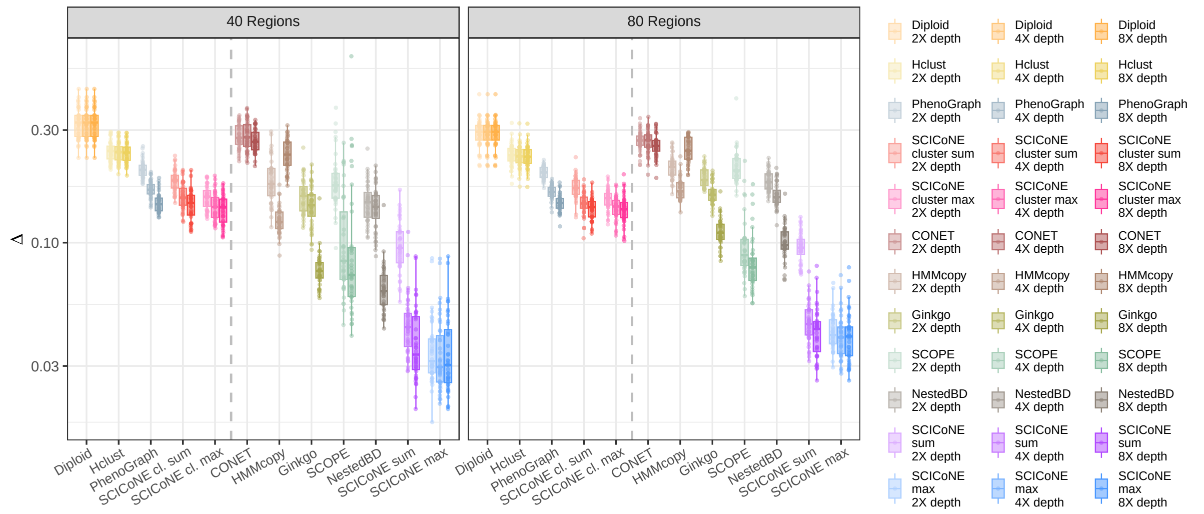

Figure S5: **Comparison of copy number calling for simulated data.** The simulation results from Figure 5 with Δ displayed on a logarithmic scale.

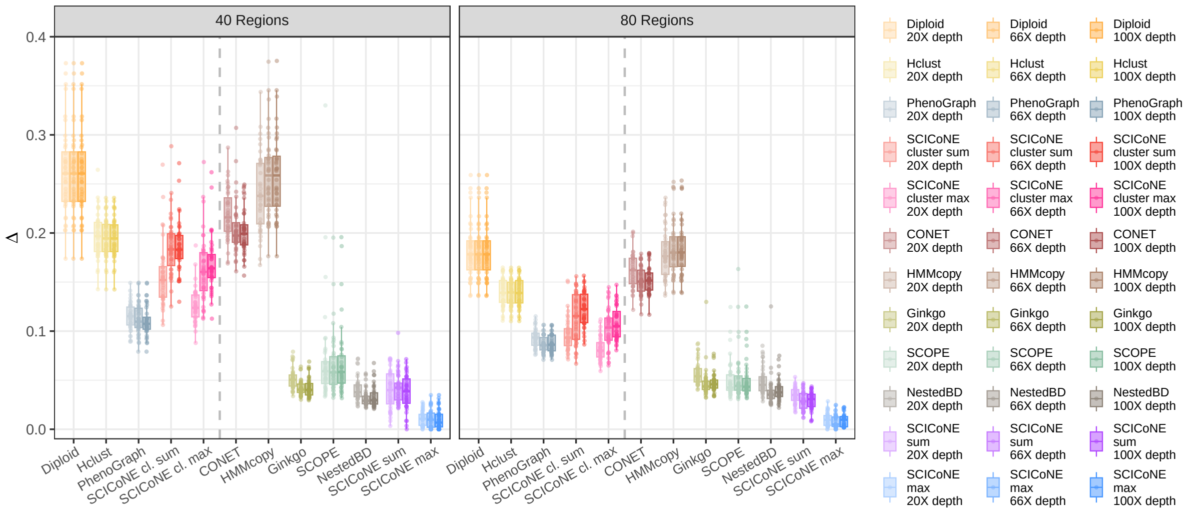

Figure S6: **Comparison of copy number calling for simulated data.** The simulation setting is as in Figure 5 but with higher coverage and without overdispersion.

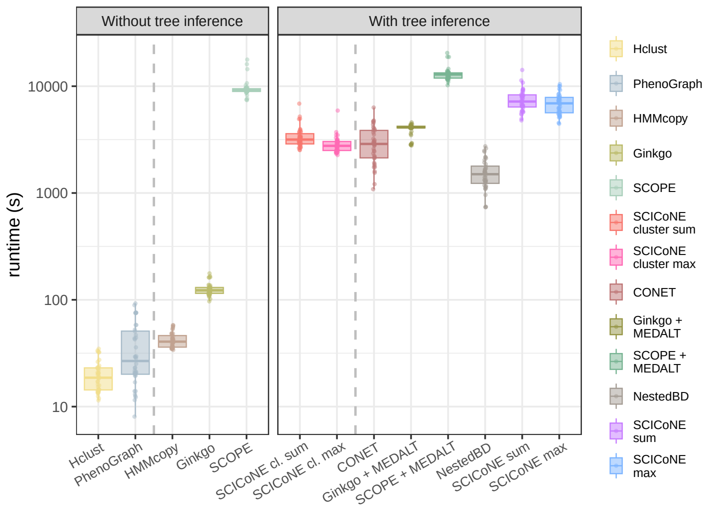

Figure S7: **Runtimes for copy number calling for simulated data.** The runtimes for the simulation setting of Figure 5 (with 4X depth and 40 regions) for the different methods. The methods in the left panel call copy numbers without reconstructing a tree, while those in the right panel also perform tree inference. Inside each panel, methods that cluster data or run on clustered data are on the left of the dashed lines.

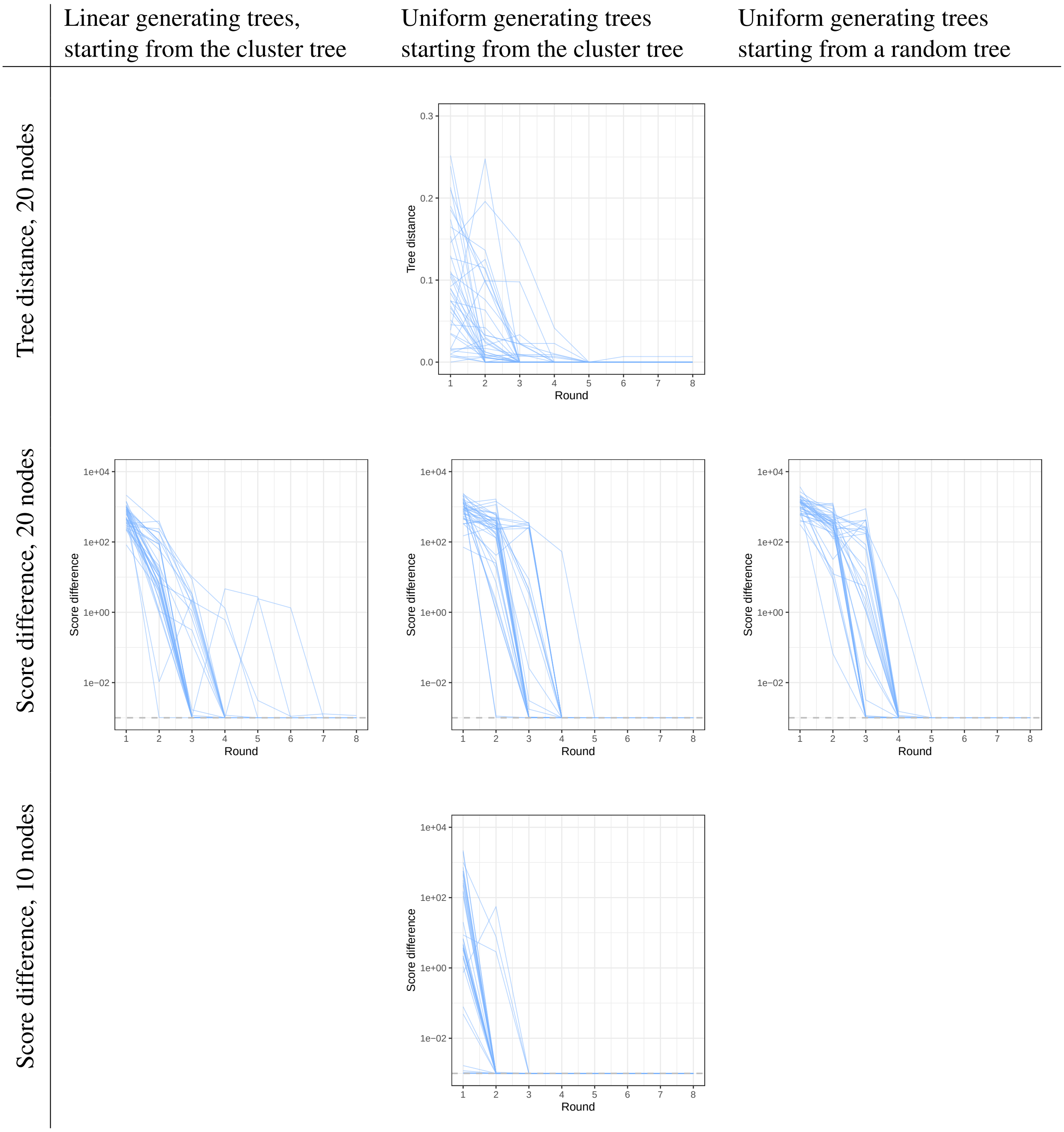

Figure S8: **Convergence of the MCMC runs.** For several rounds of shorter chains (10,000 iterations) we see convergence after a few rounds both in terms of tree distances (top row) and the scores of the trees (bottom rows). This is robust for linear trees (left column) or uniform random trees (right columns) generating the data, and whether we start from the tree inferred from the clustered data (left columns) or a random tree (right column). For smaller trees (bottom row), the convergence is notably faster. Each plot shows the results of 40 replicates.

Appendices

A Breakpoint detection

For a given bin position ρ, we wish to test if there is a change in read counts in the next bin, across cells. In particular we fix a window size ω, consider the bins from $\rho - \omega + 1$ to $\rho + \omega$ and look at the evidence for a breakpoint, and hence a copy number change, after bin ρ.

A.1 Evidence per cell For a given cell j we model the read counts in bin i, z_i^j with a negative Binomial to account for overdispersion. We parametrise in terms of the mean λ and overdispersion parameter ν with a mass function of

$$P(X = z) = \frac{\Gamma(z + \nu)}{z!\Gamma(\nu)} \left(\frac{\lambda}{\lambda + \nu}\right)^z \left(\frac{\nu}{\lambda + \nu}\right)^\nu \tag{1}$$

If there is no copy number change after bin ρ we would have equal expected counts across all bins in the window, so that the log-likelihood of the observed counts is

$$l(z^j; \lambda, \nu) = \left(\sum_{i=\rho-\omega+1}^{\rho+\omega} z_i^j\right) [\log(\lambda) - \log(\lambda + \nu)] - 2\omega\nu \log(\lambda + \nu) \tag{2}$$

where we ignore constant terms that do not depend on λ. The maximum likelihood, for fixed ν occurs at

$$\lambda^* = \frac{\sum\limits_{i=\rho-\omega+1}^{\rho+\omega} z_i^j}{2\omega} \tag{3}$$

To allow for noise giving unbalanced counts on either side of ρ even without a breakpoint, we fit a linear model for the expected counts

$$l(z^j; \alpha, \beta, \nu) = \sum_{i=\rho-\omega+1}^{\rho+\omega} z_i^j [\log(\lambda_i) - \log(\lambda_i + \nu)] - 2\omega\nu \log(\lambda_i + \nu)\,, \qquad \lambda_i = \alpha + \beta(i - \rho) \tag{4}$$

which we maximise over α and β:

$$(\alpha^*, \beta^*) = \underset{\alpha,\beta}{\text{argmax}}\ l(z^j; \alpha, \beta, \nu) \tag{5}$$

To avoid fitting real copy number changes with the linear model, we bound the slope so the relative change across ρ is less than a quarter by restricting $|\beta| < \frac{1}{4\omega}$.

If there is a breakpoint, we would expect to have different average counts on each side which we model with two mean parameters leading to a log-likelihood of

$$\begin{aligned} l(z^j; \lambda_{\mathrm{L}}, \lambda_{\mathrm{R}}, \nu) \;=\; & \left(\sum_{i=\rho-\omega+1}^{\rho} z_i^j\right) [\log(\lambda_{\mathrm{L}}) - \log(\lambda_{\mathrm{L}} + \nu)] - \omega\nu \log(\lambda_{\mathrm{L}} + \nu) \\ & + \left(\sum_{i=\rho-\omega+1}^{\rho} z_i^j\right) [\log(\lambda_{\mathrm{R}}) - \log(\lambda_{\mathrm{R}} + \nu)] - \omega\nu \log(\lambda_{\mathrm{R}} + \nu) \end{aligned} \tag{6}$$

This is maximised by the average counts of each side of the potential breakpoint: For robustness and to uncover changes in count level that spread over many bins, we use the robust mean

$$\lambda^*_{\mathrm{L}} = \mathrm{mean_r}\left(z^j_i, i = \rho - \omega + 1, \dots, \rho\right), \qquad \lambda^*_{\mathrm{R}} = \mathrm{mean_r}\left(z^j_i, i = \rho + 1, \dots, \rho + \omega\right) \tag{7}$$

We further enforce a minimum difference between the λ parameters on each side of ρ so that the relative change in copy number is more than a quarter:

$$\frac{|\lambda^*_{\mathrm{L}} - \lambda^*_{\mathrm{R}}|}{\lambda^*} > \frac{1}{4} \tag{8}$$

If the inequality is not satisfied by $\lambda^*_{\mathrm{L,R}}$, we rescale their differences from λ^*.

Finally we compute the difference in maximum log-likelihoods of the two models. We compute this difference per cell for each breakpoint

$$A^j_\rho = l(z^j; \lambda^*_{\mathrm{L}}, \lambda^*_{\mathrm{R}}, \nu) - l(z^j; \alpha^*, \beta^*, \nu) \tag{9}$$

Especially at low read-depths, the likelihood ratios over bins A^j_ρ for each cell may exhibit noise from the underlying count data. However we would expect breakpoints with a copy number change to provide a signal across the bins with a width of the window size ω. To amplify these signals and filter out noise, we perform a low-pass Gaussian filter (with half-gain cut-off frequency corresponding to twice the width ω) with a Fourier transform and replace the A^j_ρ signal per cell by the smoothed version. Since this filter may help identifying true breakpoints with high-noise, but by adding width to the signal may add uncertainly to their detected location, by default we only employ the filtering for read depths of 10 and below.

A.2 Combining cells Next we compute the logarithm of the combined evidence that the breakpoint occurred in any k of the m cells with the average

$$\Sigma^k_\rho = \log\left[\frac{1}{\binom{m}{k}} \sum_{\boldsymbol{J}\subset\{1,\dots,m\}}^{|\boldsymbol{J}|=k} \mathrm{e}^{\sum_{j\in\boldsymbol{J}} A^j_\rho}\right] \tag{10}$$

This can be computed efficiently using dynamic programming (Algorithm 1). We further combine the evidence for the breakpoint in k of the m cells with the prior of an event affecting k cells in a random binary cell lineage tree [3]:

$$P(k) = \begin{cases} (1-\mu) & k = 0 \\ \mu\frac{\binom{m}{k}^2}{(2k-1)\binom{2m}{2k}} & 1 \leq k \leq m \end{cases} \tag{11}$$

where μ is the prior probability of an event occurring. The posterior probability of the event occurring in k cells is then proportional to $P(k)\mathrm{e}^{\Sigma^k_\rho}$. To rank the breakpoints, we consider the posterior probability of the breakpoint occurring in k^* or more cells

$$P_\rho = \frac{\sum_{k=k^*}^m P(k)\mathrm{e}^{\Sigma^k_\rho}}{\sum_{k=0}^m P(k)\mathrm{e}^{\Sigma^k_\rho}} = 1 - \frac{\sum_{k=0}^{k^*-1} P(k)\mathrm{e}^{\Sigma^k_\rho}}{\sum_{k=0}^m P(k)\mathrm{e}^{\Sigma^k_\rho}} \tag{12}$$

and compute

$$S_\rho = -\log\left[1 - P_\rho\right] = \log\left[\sum_{k=0}^m P(k)\mathrm{e}^{\Sigma^k_\rho}\right] - \log\left[\sum_{k=0}^{k^*-1} P(k)\mathrm{e}^{\Sigma^k_\rho}\right] \tag{13}$$

Algorithm 1 Obtain the sum of breakpoint evidence over all cell subsets

Input The vector of penalised relative likelihoods, A_ρ^j, $j = 1, \ldots, m$
Initialise a vector v_l, $l = 0, \ldots, m$:
$v_0 = 0$
$v_1 = A_\rho^1$
for $j = 2$ to m **do** ▷ cells
  for $k = 1$ to $j - 1$ **do** ▷ size of subset
    $v'_k = \text{log.add}(v_{k-1} + A_\rho^j, v_k)$ ▷ $\text{log.add}(a, b) = \max(a, b) + \log\left[1 + \mathrm{e}^{-|a-b|}\right]$
  end for
  $v'_j = v_{j-1} + A_\rho^j$
  Update vector $v_k = v'_k$, $k = 1, \ldots, j$
end for
$\Sigma_\rho^k = v_k - \log\binom{m}{k}$, $k = 0, \ldots, m$
return Vector of summed combinations: Σ_ρ^k, $k = 0, \ldots, m$

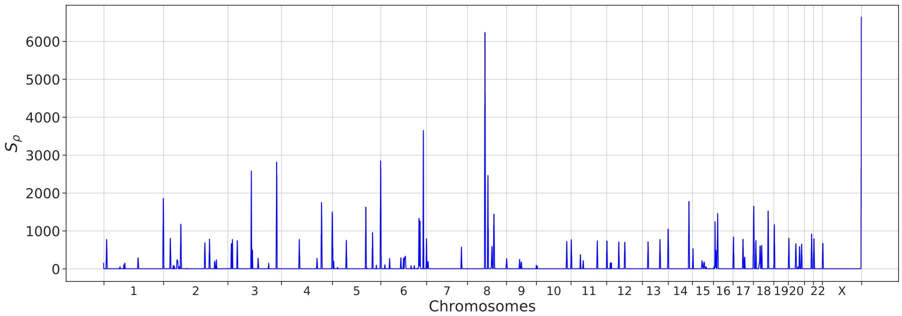

Figure S9: **Breakpoint detection** For each bin we combine the evidence for a breakpoint across the 260 cells from a breast xenograft [2], to arrive at S_ρ. Peaks corresponds to putative breakpoints.

A.3 Peak detection

Plotting S_ρ across the genome we find peaks, with a width of ω when ρ lines up with a breakpoint in a number of cells (Figure S9). Since the peaks possess very different heights, we perform a further log transform, $\tilde{S}_\rho = \log S_\rho$. Since the local median value of $\tilde{S}_\rho$ may depend on the underlying copy number state, we find the breakpoints iteratively. First we divide $\tilde{S}_\rho$ by its median value and find the highest value. If it is above the threshold, we split $\tilde{S}_\rho$ into two parts, exclude a window of size ω from either side and divide the remaining parts by their median. We repeatedly find the maximum value, split the vector excluding a window around the maximum, and divide the regions between the current breakpoints by their median until the maximum in those regions is below the threshold. The threshold has a default value of 3 times the distance between the median and third quartile of the bins not around the current set of breakpoints. We have a default window size of 20.

B Tree penalisation for combinatorial effects

When summing over the possible attachments of cells to the event tree, it is possible for the marginal likelihood to grow uncontrollably with more complex trees. In particular, for any tree, assume that the largest cluster of cells of size m_c attach best to the same node k and poorly elsewhere. The copy number state $C_i(T, V)$ at each node i is determined from the event vector V by collating all the events on the path back to the root in the tree T, which then allows us to compute the likelihood for any cell attaching

to that node (Methods). The marginal likelihood contribution of these cells includes the term

$$\prod_{j=1}^{m_c} \frac{1}{(n+1)} \sum_{\sigma_j=0}^{n} L_j(T, V, \sigma_j) \approx \frac{1}{(n+1)^{m_c}} \prod_{j=1}^{m_c} L_j(T, V, k) \tag{14}$$

where in the sum we assume that the other attachment points are negligible for the sum. Imagine we add another node k' to the tree with a very similar genotype to the best attachment point of the cells, and hence very similar likelihoods for the cells. The marginal likelihood term now becomes

$$\begin{aligned}\prod_{j=1}^{m_c} \frac{1}{(n+2)} \sum_{\sigma_j=0}^{n+1} L_j(T', V', \sigma_j) &\approx \frac{1}{(n+2)^{m_c}} \prod_{j=1}^{m_c} L_j(T, V, k) + L_j(T', V', k') \\ &\approx \frac{1}{(n+2)^{m_c}} \prod_{j=1}^{m_c} 2L_j(T, V, k) = \frac{2^{m_c}}{(n+2)^{m_c}} \prod_{j=1}^{m_c} L_j(T, V, k)\end{aligned} \tag{15}$$

so that compared to before it can be increased by a exponential factor of $\left[\frac{2(n+1)}{(n+2)}\right]^{m_c}$. The likelihood contribution can therefore be made arbitrarily large by adding further dummy nodes with similar genotypes. Since the exponential increase is bound by 2^{m_c}, to counteract this effect, we can penalise trees with the factor

$$P'(T) = \frac{1}{2^{nm_c}} \tag{16}$$

so that if we add an additional node and increase n by 1 we obtain an additional factor of 2^{-m_c}. This ensures that the marginal likelihood cannot be increased by adding additional nodes to the tree which just essentially recreate genotypes that are already present.

Note that when we maximise and take the best attachment point per cell, rather than summing over all attachment points to marginalise, the best score does not increase by having additional attachment points. For the example above, the maximum likelihood contribution includes the term

$$\prod_{j=1}^{m_c} \max_{0 \leq \sigma_j \leq n+1} L_j(T, V, \sigma_j) = \prod_{j=1}^{m_c} L_j(T, V, k) \tag{17}$$

which remains unchanged when we add another node k' with similar (but lower) likelihood

$$\prod_{j=1}^{m_c} \max_{0 \leq \sigma_j \leq n+2} L_j(T, V, \sigma_j) = \prod_{j=1}^{m_c} \max\left\{L_j(T, V, k), L_j(T, V, k')\right\} = \prod_{j=1}^{m_c} L_j(T, V, k) \tag{18}$$

so we do not need to include additional penalisation.

C Prior on the event vector

For the prior $P(V \mid T)$ for the event vector V with a given tree T, we simply consider that each amplification or deletion may be selected among the K segments with a choice of sign, leading to a factor of $\frac{1}{2K}$. At each node, we consider the amplification of contiguous segments as a single amplification event, and likewise for deletions. To compute the number of contiguous events, let us list as $\boldsymbol{v}^i$ a vector of the number of copy number events for each segment occurring at node i in the tree. For the example in Figure 1c with event vector $V = (+S_1 + S_2,\ +S_2 + S_3,\ -S_1,\ -S_4,\ +S_2)$ we would have $\boldsymbol{v}^2 = (0, +1, +1, 0, 0)$ over the $K = 5$ segments. To count the number of contiguous amplifications and deletions we can simply count when the copy number profile increases as

$$|v^i| = \sum_{k=1}^{K+1} \left(v_k^i - v_{k-1}^i\right) I\left(v_k^i - v_{k-1}^i > 0\right), \qquad v_0^i = v_{K+1}^i = 0 \tag{19}$$

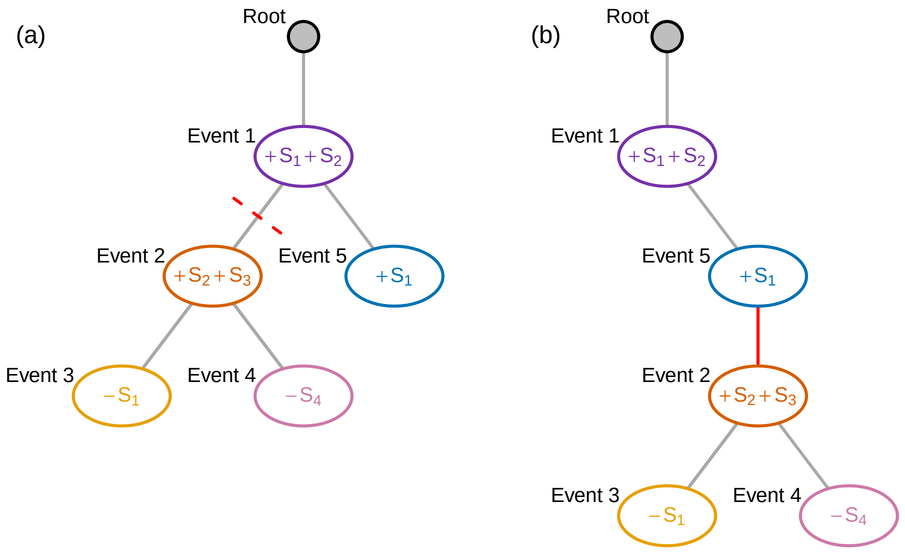

Figure S10: **Prune and reattach.** From the event tree in (a), we propose the new tree in (b) with the *prune and reattach* move by sampling a node (event node 2), detaching it from the tree and sampling a new parent (node 5).

where I is the indicator function. The prior probability for each node is then $\frac{1}{(2K)^{|v^i|}}$.

For the full event vector prior, we may freely permute the ordering of the event vector, along with the corresponding numbering of the event nodes in the tree. We therefore divide by the overcounting factor of $n!$ to arrive at

$$P(V \mid T) = \frac{1}{n!} \prod_{i=1}^{n} \frac{1}{(2K)^{|v^i|}} \tag{20}$$

Note that although the overcounting factor assumes that the elements of the event vector are distinct, we keep the same value in all cases for simplicity.

As a further penalisation, whenever an event has the opposite sign to the cumulative copy number state of the parent node, we subtract α from the log-likelihood, for which we have the default value $\alpha = 10$.

D MCMC moves

Prune and reattach The most basic move to change trees with a fixed event vector is *prune and reattach*. From the current tree T, we propose a new tree T' by uniformly sampling a node (except the root), detaching it and its descendant subtree and then uniformly sampling a new parent from the remainder of the tree including the root. An example of this proposal move is depicted in Figure S10. After the new tree has been proposed, we simply accept the move with probability

$$\rho = \min\left\{1, \frac{P(T', V \mid D) Q(T \mid T')}{P(T, V \mid D) Q(T' \mid T)}\right\} \tag{21}$$

where $Q(T' \mid T)$ is the probability of proposing tree T' when currently at tree T in the chain. Since the move is symmetric, $Q(T' \mid T) = Q(T \mid T')$, the acceptance ratio simplifies to

$$\rho = \min\left\{1, \frac{P(T', V \mid D)}{P(T, V \mid D)}\right\} \tag{22}$$

Since only the detached subtree needs to be rescored, it is cheaper to move smaller subtrees and more of such moves can be processed for the same computational cost. To try and speed up convergence of the MCMC scheme, we can preferentially sample nodes to move which have fewer descendants. We denote by $d_i(T)$ the size of the subtree of T starting with node i (the number of descendants plus 1). Instead of uniformly sampling nodes, we can sample proportionally to the $d_i(T)^{-1}$ to balance the computational cost of different moves.

If we define

$$\zeta(T) = \sum_{i=1}^{n} d_i(T)^{-1} \tag{23}$$

as a normalising constant then the transition probabilities in moving from tree T to T' where node i is selected to detach are then

$$Q(T' \mid T) = \frac{d_i(T)^{-1}}{\zeta(T)}, \qquad Q(T \mid T') = \frac{d_i(T')^{-1}}{\zeta(T')}, \qquad \frac{Q(T \mid T')}{Q(T' \mid T)} = \frac{\zeta(T)}{\zeta(T')} \tag{24}$$

and the acceptance probability becomes

$$\rho = \min\left\{1, \frac{P(T', V \mid D)\zeta(T)}{P(T, V \mid D)\zeta(T')}\right\} \tag{25}$$

Swap node labels A further move is to select two nodes uniformly at random and swap over the events associated with each node. All nodes below either affected node need to be rescored.

This move is symmetric and hence accepted with probability

$$\rho = \min\left\{1, \frac{P(T', V \mid D)}{P(T, V \mid D)}\right\} \tag{26}$$

However, again we create a weighted version. We define $e_{ij}(T)$ to be the number of nodes affected by the swap. This is the number of descendants of node i plus the number of descendants of node j plus 2, if i and j are in different lineages, or the number of descendants of the ancestor minus the number of descendants of the lower node if they are in the same lineage. Then we can sample the pair (i, j) proportionally to $e_{ij}(T)^{-1}$. Since the tree structure does not change however, this move is also symmetric.

Add or remove events This move changes the event vector, but keeps the tree fixed. We first select a node at random (uniformly). Then we sample the number of segments to be affected from a $\mathrm{Poisson}(\lambda_R) + 1$ and sample this number of (distinct) segments uniformly. Then for each segment we sample the number of additional copies from a $\mathrm{Poisson}(\lambda_C) + 1$ and a sign uniformly. If the change leads to all events at the node being completely cancelled (say at node 3 in Figure S10a which only has the event $-S_1$ we sample adding $+S_1$), then we simply reject the move.

This move is symmetric (since the sign accounts for adding and deleting) so the acceptance ratio is

$$\rho = \min\left\{1, \frac{P(T, V' \mid D)}{P(T, V \mid D)}\right\} \tag{27}$$

Setting $\lambda_C = 0$ and $\lambda_R = 0$ makes this move select only a single segment, and only allows its number of copies to change by 1.

In the weighted version, we sample nodes proportionally to $d_i(T)^{-1}$ as for the weighted prune and reattach, but since the tree structure does not change, the move is still symmetric.

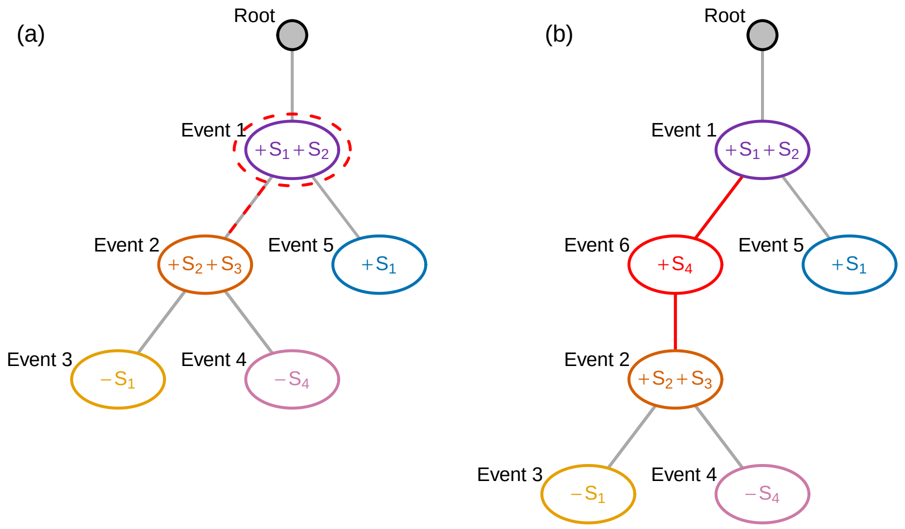

Figure S11: **Add or remove node.** From the tree in (a) [which is the tree in Figure S10a] we sample to add a new node and select event 1 as the new parent. From the two children of event node 1, we select event node 2 to become a child of the newly inserted node. With the node inserted, we sample the events in the new node as an amplification of segment 4 to arrive at the tree in (b).

Add or remove node This move changes both the tree structure and the event vector, as in the example of Figure S11. To define the move, we first consider the possible ways of adding a node. We may place the new node below any of the $(n+1)$ nodes in the current tree including the root. However, when we place the new node below a node with δ children, any of the δ may become children of the new node instead of remaining siblings. There are therefore

$$\chi(T) = \sum_{i=0}^{n} 2^{\delta_i(T)} \tag{28}$$

possible placements of the new node in the tree T where δ_i is the number of children of node i. For the example in Figure S11a then $\delta = (1, 2, 2, 0, 0, 0)$ and hence $\chi = 13$.

For the new node, we sample the number of segments to be affected with a $\text{Poisson}(\lambda_R) + 1$. For each of the r segments we select from the K segments in total with $\binom{K}{r}$ ways. If $r > K$ we just reject the move for simplicity. Then for each of the selected segments we sample a sign uniformly and a number of copies c from $\text{Poisson}(\lambda_C) + 1$. The transition probability, given that we selected to add a node, to that exact tree with that exact label is then

$$Q_{\text{add}}(T' \mid T) = \frac{\lambda_R^{(r-1)} \mathrm{e}^{-\lambda_R} \lambda_C^{\sum_{j=1}^{r}(c_j-1)} \mathrm{e}^{-r\lambda_C}}{2^r \binom{K}{r} \chi(T)(r-1)! \prod_{j=1}^{r}(c_j-1)!} \tag{29}$$

Since there are many more ways to add a labelled node, than to delete one, we weight the deletion by the corresponding terms in adding the node back. For example, for each node i of the n nodes to delete, we count the number of segments r_i and the number of copies $c_j, j = 1, .., r_i$ and weight that node by the factor

$$w_i(T, V) = \frac{\lambda_R^{(r_i-1)} \mathrm{e}^{-\lambda_R} \lambda_C^{\sum_{j=1}^{r_i}(c_j-1)} \mathrm{e}^{-r_i\lambda_C}}{2^{r_i} \binom{K}{r_i}(r_i-1)! \prod_{j=1}^{r_i}(c_j-1)!} \tag{30}$$

with the sum of these deletion weights, $W(T,V) = \sum_{i=1}^{n} w_i(T,V)$.

For the move, we first sample, with equal probability, whether to delete or add. If delete is chosen then we sample a node from the n available proportionally to the weights $w_i(T,V)$, with probability $\frac{w_i(T,V)}{W(T,V)}$. The sampled node is removed and all of its children are attached to the nodes previous parent. If add is chosen, we sample a node from the $(n+1)$ available including the root proportionally to $2^{\delta_i(T)}$. The new node is inserted as a child of the sampled node. The previous children of the sampled node are each independently randomly assigned to become children of the new inserted node with probability $\frac{1}{2}$, otherwise they remain siblings of the new inserted node. Finally the label of the inserted node is sampled with the Poisson distributions for the number of segments affected, their numbers of copies and uniformly chosen signs for each.

Since the weights for deletion include all the terms corresponding to sampling, for detailed balance we accept the move with a probability of

$$\begin{aligned} \rho_{\text{add}} &= \min\left\{1, \frac{P(T',V' \mid D)\chi(T)}{P(T,V \mid D)W(T',V')}\right\} \\ \rho_{\text{del}} &= \min\left\{1, \frac{P(T',V' \mid D)W(T,V)}{P(T,V \mid D)\chi(T')}\right\} \end{aligned} \tag{31}$$

depending on whether add or delete was selected.

For weighting the moves also according to their computational cost, we need to further weight each term in the proposal neighbourhood by the cost of updating the score. For each node i to be deleted there are a remaining $(d_i(T) - 1)$ nodes to rescore so we define a new weight (without the minus 1)

$$u_i(T,V) = \frac{w_i(T,V)}{d_i(T)}, \qquad U(T,V) = \sum_{i=1}^{n} u_i(T,V) \tag{32}$$

For adding nodes, say the new parent has δ children, we would need to compute the weights for all 2^δ choices. For simplicity we weight all those choices by assuming that on average only half of its descendants are affected along with the new node. The weights for adding are

$$\xi_i(T) = \frac{2^{\delta_i(T)+1}}{d_i(T)+1}, \qquad \Xi(T) = \sum_{i=0}^{n} \xi_i(T) \tag{33}$$

and the acceptance probability becomes

$$\begin{aligned} \rho_{\text{add}} &= \min\left\{1, \frac{P(T',V' \mid D)\Xi(T)2d_{i'}(T')}{P(T,V \mid D)U(T',V')(d_i(T)+1)}\right\} \\ \rho_{\text{del}} &= \min\left\{1, \frac{P(T',V' \mid D)U(T,V)(d_{i'}(T')+1)}{P(T,V \mid D)\Xi(T')2d_i(T)}\right\} \end{aligned} \tag{34}$$

Condense or split nodes Finally we allow nodes to be split into two, or to combine a parent and child into one, as in the example of Figure S12.

We first consider the move where we split a single node into a parent-child. If we select the node i we would look at the vector $\boldsymbol{v}^i$ of the copy number events at that node. Let's say we have the vector $\boldsymbol{v} = (0, -1, 2, 0, 0)$ which we need to split into two vectors. We sample the splits with a Poisson

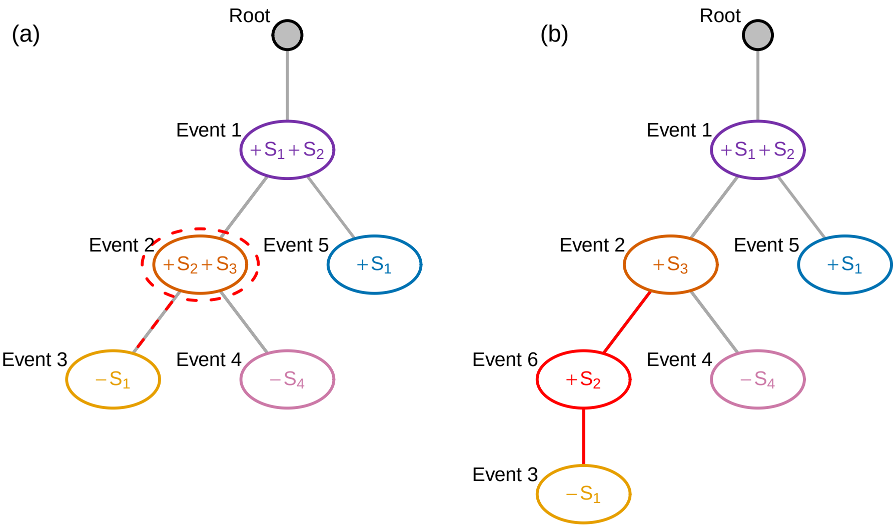

Figure S12: **Condense or split nodes.** From the tree in (a) [which is again the tree in Figure S10a] we sample event node 2 to split. From its two children, we select event node 3 to become a child of the newly split off node which becomes event node 6. With the node placed, we sample how to split the events which were previously at event node 2 across that node and its new child to arrive at the tree in (b).

distribution with parameter λ_S in the following way:

$$\boldsymbol{v}_k^p = \begin{cases} \alpha\beta\Lambda & \boldsymbol{v}_k = 0 \\ \frac{\boldsymbol{v}_k}{2} + \beta\left[\frac{1}{2} + \Lambda\right] & \boldsymbol{v}_k \text{ odd} \\ \frac{\boldsymbol{v}_k}{2} + \beta\Lambda & \boldsymbol{v}_k \text{ even} \end{cases}, \quad \begin{array}{lcl} \alpha & \sim & \text{Bernoulli}\left(\alpha_0\right) \\ \beta & \sim & 2\text{Bernoulli}\left(\frac{1}{2}\right) - 1 \\ \Lambda & \sim & \text{Poisson}(\lambda_S) \end{array} \tag{35}$$

where for positions which are 0 in $\boldsymbol{v}$ we allow them to be split into cancelling events with a low probability α_0. This sampling provides the proposed event vector of the parent, while that of the child is fixed by $\boldsymbol{v}^p + \boldsymbol{v}^c = \boldsymbol{v}$. If either vector ends up being completely 0, we reject the move.

For each of the segments we compute the absolute difference c between the number of copies in the new parent vector and the new child vector. The probability of picking each difference is

$$f(c) \underset{\boldsymbol{v}_k \neq 0}{=} \begin{cases} \mathrm{e}^{-\lambda_S} & c = 0 \\ \frac{\lambda_S^{\frac{c-1}{2}} \mathrm{e}^{-\lambda_S}}{2(\frac{c-1}{2})!} & c \text{ odd} \\ \frac{\lambda_S^{\frac{c}{2}} \mathrm{e}^{-\lambda_S}}{2(\frac{c}{2})!} & c \text{ even} \end{cases}, \qquad f(c) \underset{\boldsymbol{v}_k = 0}{=} \begin{cases} (1-\alpha_0) + \alpha_0 \mathrm{e}^{-\lambda_S} & c = 0 \\ \alpha_0 \frac{\lambda_S^{\frac{c}{2}} \mathrm{e}^{-\lambda_S}}{2(\frac{c}{2})!} & c \text{ even} \end{cases} \tag{36}$$

For the split, we do not consider the root so there are

$$\chi(T, V) = \sum_{i=1}^{n} 2^{\delta_i(T)} \tag{37}$$

ways of arranging splits. The transition probability to that exact tree and exact event vector is

$$Q_{\text{split}}(T', V' \mid T, V) = \frac{\prod_{j=1}^{r} f(c_j)}{\chi'(T, V)} \tag{38}$$

To compensate for this we weight the reverse move of combining a node with its parent by

$$w_i(T, V) = \prod_{j=1}^{r} f(c_j) I(\mathrm{Pa}_i \neq 0) \tag{39}$$

where the c_j are the absolute difference in copy number change in the non-zero segments in the node and its parent. Nodes attached to the root get a weight of 0, which we denote with the indicator function I on the parent Pa_i of node i where 0 represents the root.

The rest of the move follows analogously to the *add or remove node* move, including the weighted version.

Genotype-preserving prune and reattach Finally we introduce a variant of *prune and reattach* where instead of retaining the event vector at the pruned node, we retain its genotype of the full copy number profile at that attachment point. When the node is reattached, the event vector of the pruned node is simply updated as the difference in the previous genotype of the pruned node and that of the new attachment point. In the example of Figure S10a the total genotype at event node 2 is $(+S_1 + 2S_2 + S_3)$ while if we were to reattach below event node 5 with genotype $(+2S_1 + S_2)$ we would replace the event vector at event node 2 by $(-S_1 + S_2 + S_3)$. As the copy number states at each event node are preserved, the likelihood of the current and proposed tree are identical and only the proposed event vector prior needs to be computed and compared to the current value to decide on the acceptance of the move.

Since computing the score of a proposal just involves evaluating the event prior, for this move we score the entire neighbourhood of prune and reattach proposals (including the current tree), and sample directly from the neighbourhood.

Changing the overdispersion Alongside tree moves, we also change the overdispersion parameter ν. Since this parameter is positive we work on the log space and propose a new value ν'

$$\log(\nu') = \log(\nu) + \epsilon\,, \qquad \epsilon \sim \mathcal{N}(0, \sigma_\epsilon^2) \tag{40}$$

using a Gaussian random walk. The standard deviation of the walk can be adapted to the standard deviation of the recent $\log(\nu)$ values of the chain [with a minimum]. The move to ν' is accepted with probability

$$\rho = \min\left\{1, \frac{P(T, V, \nu' \mid D)}{P(T, V, \nu \mid D)}\right\} \tag{41}$$

since the move is symmetric.

Adaptive tempering To speed up the search procedure for the highest scoring tree, we can raise the score to a power $P(T, V \mid D)^\gamma$, and adjust the likelihood landscape by varying γ. For $\gamma > 1$ we amplify the differences between tree scores and spend more time exploring a local neighbourhood, while for $\gamma < 1$ we flatten the landscape and move globally more easily. We vary γ adaptively by keeping track of the acceptance probability of moves in the chain. For every tree move which is accepted we transform $\gamma \to \gamma \mathrm{e}^{a(1-a)}$ and for every move that is rejected $\gamma \to \gamma \mathrm{e}^{-a^2}$ so that we aim to keep the acceptance probability at a. As a default we set $a = \frac{1}{K}$ as with more segments it is less likely to pick one that improves the tree.

Maximisation instead of summation Instead of the summation of Equation (9), we can target the score defined by attaching cells at the maximally scoring placement using Equation (10) which avoids the need to penalise the combinatorial complexity. In the scheme above, we simply replace $P(T, V \mid D)$

everywhere by $S(T, V \mid D)$ and search for the maximal score S. Since we no longer need to satisfy detailed balance, we simplify the acceptance probability of each move to

$$\rho = \min\left\{1, \frac{S(T', V \mid D)}{S(T, V \mid D)}\right\}. \tag{42}$$

Extract common ancestor In the maximisation mode, we additionally propose to select a pair of sibling nodes uniformly at random, identify the set of common events between them, remove those from each node in the pair, and add them in a new node that is inserted as their new parent. This does not affect the genotypes of the pair of nodes, but may provide a more parsimonious event history. Since the move is not reversible on its own, we preclude it from the sampling scheme and employ it for maximisation.

Expand or shrink event In the maximisation mode, we also include an extension of the *add or remove events* move in which we expand or shrink existing event blocks. We first select a node uniformly at random. Then we choose an event block of a node selected uniformly at random, select either its start or end, and then either remove the event at the edge or expand it to the next contiguous region. Since expanding blocks may merge with others, the reversibility of the move could be quite involved and for simplicity we employ this move only for maximisation.

E Detailed comparison with CONET

In the main simulation results (Figure 5), CONET performs poorly compared to all other methods, and worse than the results reported in their paper [4]. Indeed, when we run CONET on the simulated data with 2X read depth per bin, it typically finds a large tree with each bin having its own events and does not find the true copy number signal. At these very low coverages, CONET does not have much information from the breakpoint count differences which it relies on for its likelihood computations and tree inference. To recreate results similar to those reported for CONET, we consider their simulation scheme whereby normalised single-cell whole-genome sequencing read depths were generated from copy number profiles by adding Gaussian noise with copy-number-specific variance. In their low-noise setting, these variances are (0.2, 0.01, 0.03, 0.01, 0.07) for copy number values of (0, 1, 2, 3, 4), respectively, and they are doubled for the high-noise case. CONET also assumes a Gaussian noise model for the corrected read counts in the inference.

Since read count data is naturally discrete, this Gaussian approximation corresponds to rescaling discrete data to a mean level of 2 for diploid cells. Since a Gaussian model can also correspond to having no overdispersion, we first compute the effective coverage needed for a discrete model to match the noise in their simulations [4]. For a diploid cell, the coefficient of variation (ratio between standard deviation and mean) is $\frac{\sqrt{0.03\times2}}{2} \approx 0.122$ in the high-noise setting, and to get the same variation from a Binomial distribution (as the marginal distribution of each bin) would require

$$\frac{\sqrt{0.03 \times 2}}{2} = \frac{\sqrt{\frac{N}{B}\left(1 - \frac{1}{B}\right)}}{\frac{N}{B}} \tag{43}$$

where N is the total number of reads, B the number of bins with the probability of a read landing in the bin being $\frac{1}{B}$, and $\frac{N}{B}$ is the per-bin coverage. Solving for the coverage (assuming a large number of bins) we find

$$\frac{N}{B} \approx \frac{2}{0.03} \approx 66 \tag{44}$$

so that the simulation setting of [4] corresponds to coverage of around 66X for their high-noise case and

twice as high in their low-noise setting. Any overdispersion in the data generating process would also correspond to even higher coverage.

Although these coverages are much higher than in our simulation (Figure 5), and read depths of very shallow single-cell whole genome sequencing as in [5], they are comparable to that of the datasets of [2, 6]. We therefore repeat our simulations at higher coverages and without overdispersion (Supplementary Figure S6). Although CONET starts to improve at the higher coverages, and gives results closer to their reported ones (bottom row of Figure 3 of [4]) its performance is still worse than Ginkgo, SCOPE and SCICoNE. SCICoNE even starts to give perfect reconstruction in 10-15% of cases.

Though we can come closer to CONET's previously reported performance by upping the coverage, the authors of CONET [4] claim to outperform SCICoNE, which is not the case in our simulations. One potential source of errors is that SCICoNE works directly with the read count data, and assumes a Dirichlet-multinomial distribution of the read counts per bins which allows for some overdispersion. However, the authors of [4] gave to SCICoNE not the actual read counts per bin, but normalised versions rescaled to 2 for diploid regions. When this data is used as input to SCICoNE, it will assume the average per-bin read depth is therefore around 2, and the likelihood component of SCICoNE will be artificially damped down by a large factor.

To illustrate this, we generate data according to the Gaussian simulation model of CONET [4], where the random trees are uniformly sampled apart from a linear trunk which comprises a uniform random fraction between 10% and 40% of the nodes. We use their code for generating the simulated trees and data. At these high coverages, we can detect breakpoints without a sliding window and set the window size to a single bin.

For regions with copy number state 0, for which we might expect no reads, the default standard deviation of around 0.45 (variance of 0.2) produces a lot of reads, so we also consider a lower standard deviation of 0.045. As a default we use a very low $\eta = 10^{-4}$ to model regions with no copies so that reads in those regions should be unlikely. To make the comparison more sensible, we set the value η for SCICoNE to 0.4 for both standard deviations. When SCICoNE is given as input raw read-count data, we see a distinct improvement in the performance of SCICoNE over CONET (Supplementary Figure S13a). However, when SCICoNE is given the normalised read counts as input instead of the raw counts its performance suffers greatly (Supplementary Figure S13a) since the model is being provided with artificially lowered coverage, and treats this input as observed read counts. Additionally, at such low supposed coverage, we require a sliding window to be able to detect breakpoints and set the window size to 20. The artificially worsened results in Supplementary Figure S13a are very much in line with those reported in the comparisons of [4], while the results here show that by simply giving SCICoNE the correct input it outperforms CONET, in line with our results in the main paper. These results extend to the tree distance measure (Supplementary Figure S13b) though here the 'sum' version of SCICoNE is closer to CONET, with a wider spread, while the 'max' version of SCICoNE clearly offers a distinct improvement.

These simulations favour CONET since their data simulator matches their modelling and inference scheme. However, we can further disadvantage SCICoNE by using the default value of $\eta = 10^{-4}$ so that we have a large mismatch between the simulated number of reads under the CONET model for regions with no copies and the expected number from this default in SCICoNE. Even with such heavy model misspecification, SCICoNE still outperforms CONET (Supplementary Figure S14), and is easy to improve further (as in Supplementary Figure S13) by simply adjusting η to more sensible values for the simulation setting.

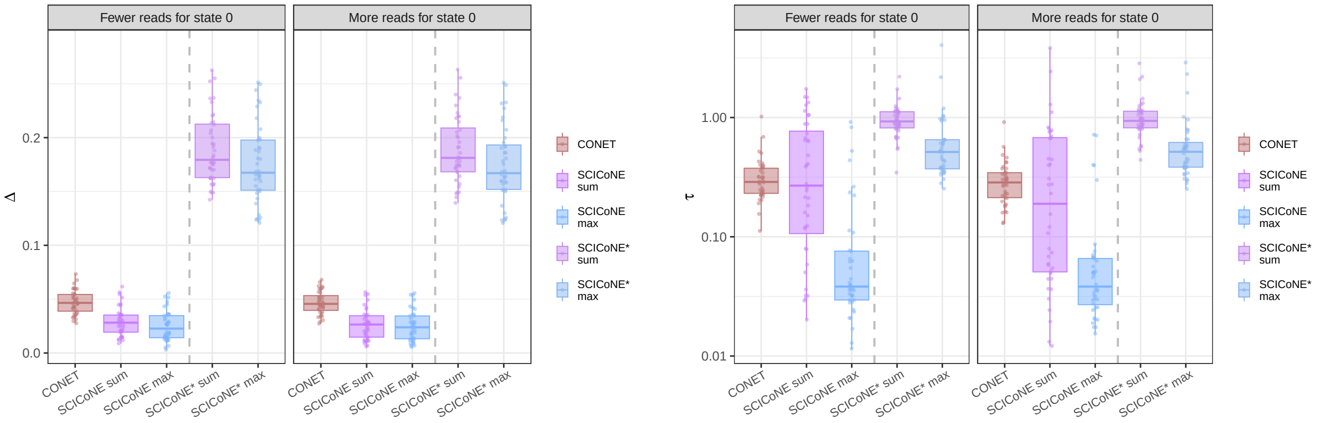

(a) (b)

Figure S13: **Comparison of copy number calling and tree reconstruction for CONET simulated data.** We generate data as in [4] and run both CONET and SCICoNE. Additionally, we run SCICoNE with normalised read data rather than read counts, indicated by the asterisk, with results on the right hand side of the plots. (a) Comparison in terms of copy number calling. (b) Comparison in terms of the tree distance τ (Methods) between the true and inferred tree for both CONET and SCICoNE.

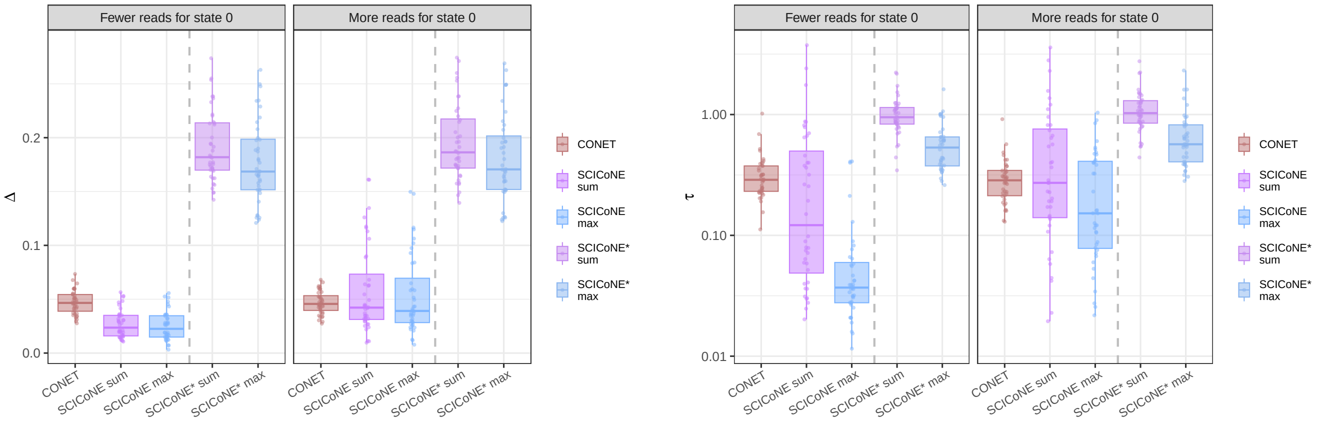

(a) (b)

Figure S14: **Comparison of copy number calling and tree reconstruction for CONET simulated data.** The comparison is as in Supplementary Figure S13 but we use the default SCICoNE model with a very low number of expected reads in regions with no copies and the parameter value $\eta = 10^{-4}$, which is strongly misspecified compared to the data generating process.

References

[1] Campbell, K. R. *et al.* clonealign: statistical integration of independent single-cell RNA and DNA sequencing data from human cancers. *Genome Biology* **20**, 54 (2019).

[2] Zahn, H. *et al.* Scalable whole-genome single-cell library preparation without preamplification. *Nature Methods* **14**, 167 (2017).

[3] Singer, J., Kuipers, J., Jahn, K. & Beerenwinkel, N. Single-cell mutation identification via phylogenetic inference. *Nature Communications* **9**, 5144 (2018).

[4] Markowska, M. *et al.* CONET: Copy number event tree model of evolutionary tumor history for single-cell data. *Genome Biology* **23**, 1–35 (2022).

[5] Irmisch, A. *et al.* The Tumor Profiler Study: integrated, multi-omic, functional tumor profiling for clinical decision support. *Cancer Cell* **39**, 288–293 (2021).

[6] Laks, E. *et al.* Clonal decomposition and DNA replication states defined by scaled single-cell genome sequencing. *Cell* **179**, 1207–1221 (2019).
